# Reverse Screening Methods to Search for the Protein Targets of Chemopreventive Compounds

**DOI:** 10.3389/fchem.2018.00138

**Published:** 2018-05-09

**Authors:** Hongbin Huang, Guigui Zhang, Yuquan Zhou, Chenru Lin, Suling Chen, Yutong Lin, Shangkang Mai, Zunnan Huang

**Affiliations:** ^1^Key Laboratory for Medical Molecular Diagnostics of Guangdong Province, Dongguan Scientific Research Center, Guangdong Medical University Dongguan, China; ^2^The Second School of Clinical Medicine, Guangdong Medical University Dongguan, China; ^3^School of Pharmacy, Guangdong Medical University Dongguan, China

**Keywords:** drug design, reverse screening, shape similarity, pharmacophore modeling, reverse docking, methodology, online service, screening databases

## Abstract

This article is a systematic review of reverse screening methods used to search for the protein targets of chemopreventive compounds or drugs. Typical chemopreventive compounds include components of traditional Chinese medicine, natural compounds and Food and Drug Administration (FDA)-approved drugs. Such compounds are somewhat selective but are predisposed to bind multiple protein targets distributed throughout diverse signaling pathways in human cells. In contrast to conventional virtual screening, which identifies the ligands of a targeted protein from a compound database, reverse screening is used to identify the potential targets or unintended targets of a given compound from a large number of receptors by examining their known ligands or crystal structures. This method, also known as *in silico* or computational target fishing, is highly valuable for discovering the target receptors of query molecules from terrestrial or marine natural products, exploring the molecular mechanisms of chemopreventive compounds, finding alternative indications of existing drugs by drug repositioning, and detecting adverse drug reactions and drug toxicity. Reverse screening can be divided into three major groups: shape screening, pharmacophore screening and reverse docking. Several large software packages, such as Schrödinger and Discovery Studio; typical software/network services such as ChemMapper, PharmMapper, idTarget, and INVDOCK; and practical databases of known target ligands and receptor crystal structures, such as ChEMBL, BindingDB, and the Protein Data Bank (PDB), are available for use in these computational methods. Different programs, online services and databases have different applications and constraints. Here, we conducted a systematic analysis and multilevel classification of the computational programs, online services and compound libraries available for shape screening, pharmacophore screening and reverse docking to enable non-specialist users to quickly learn and grasp the types of calculations used in protein target fishing. In addition, we review the main features of these methods, programs and databases and provide a variety of examples illustrating the application of one or a combination of reverse screening methods for accurate target prediction.

## Introduction

New drugs can be designed via traditional receptor structure-based virtual screening, which enables the discovery of bioactive compounds that bind the target protein, but they can also originate from reverse virtual screening, which finds the unknown protein targets of active compounds or additional targets of existing drugs (drug repositioning; Hurle et al., 2013). Among the 84 drug products introduced to the market in 2013, new indications of existing drugs accounted for 20%, implying that drug repositioning plays a key role in drug discovery (Graul et al., 2014; Li J. et al., 2016). The majority of drugs or bioactive compounds exert their functions by interacting with protein targets. With an increasing number of drugs showing the ability to target multiple proteins, target identification plays an important role in the fields of drug discovery and biomedical research (Wang J. et al., 2016). Many reverse screening methods can be used to search for the protein targets of molecules (Ziegler et al., 2013), although the earliest approaches involved expensive and time-consuming biological assays (Drews, 1997). However, with the continuous development of Big Data and computational techniques, computer-aided reverse screening methods are playing an increasingly important role in the prediction of the off-target effects and side effects of drugs as well as in drug repositioning (Rognan, 2010; Liu et al., 2014; Schomburg and Rarey, 2014).

**Figure 1:**
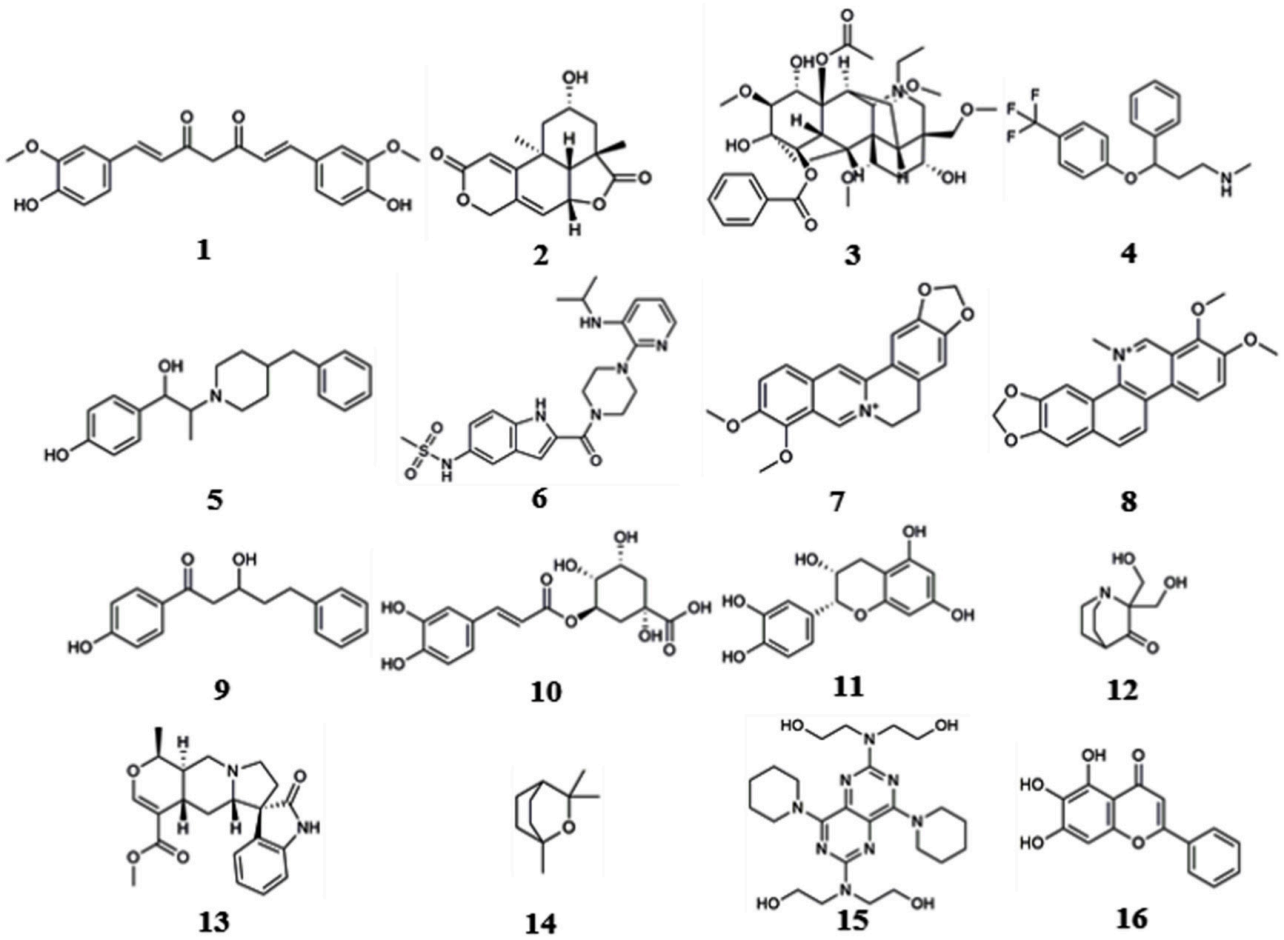
Compounds described in the manuscript.

These computational methods can be divided into three classes according to their underlying principles: shape screening, pharmacophore screening, and reverse docking. In the absence of receptor crystal structures, shape or pharmacophore screening facilitates the discovery of the potential targets of a query molecule by comparing its overall shape or key pharmacophore features with those of the compounds from a ligand database annotated with target information (Schuffenhauer et al., 2003; Hawkins et al., 2007; Chen et al., 2009). The annotated targets of the matched ligands can then be considered potential targets of the query molecule. Reverse docking, in contrast to the traditional molecular docking used to find the ligands of a target protein, refers to the successive docking of a query molecule into the active pocket of each protein from a protein 3D structure database based on spatial and energy principles to identify protein targets with strong binding affinity as potential targets of the query molecule (Li et al., 2013). Reverse screening methods are important computational techniques for identifying new macromolecular targets of existing drugs or active molecules and for analyzing their functional mechanisms or side effects (Patel et al., 2015). Based on the principles of the methods and the availability of existing large-scale small-molecule [e.g., ChEMBL, the European Molecular Biology Laboratory (Gaulton et al., 2017)] or macromolecule (e.g., the PDB; Rose et al., 2015) databases, researchers worldwide have developed a variety of software and online services for predicting the protein targets of small molecules. Representative examples include SEA (Keiser et al., 2007), PharmMapper (Liu et al., 2010) and INVDOCK (Chen and Zhi, 2001), which are among the earliest tools for shape screening, pharmacophore screening and reverse docking, respectively. In recent years, these three methods have been widely used in the prediction of protein targets to clarify the molecular mechanisms of active small molecules against various diseases (Kharkar et al., 2014; Cereto-Massagué et al., 2015). Many of these molecules are derived from Chinese herbal medicine, and while their pharmacological or biological activities are known, their cellular and molecular mechanisms remain unclear. For example, Lim et al. (2014) used shape screening to determine that curcumin (compound **1**, Figure 1), extracted from Zingiberaceae, suppresses the proliferation of human colon cancer cells by targeting cyclin dependent kinase 2 (CDK2). Marine compounds are another class of bioactive small molecules. For example, wentilactone B (WB, compound **2**) is a tetranorditerpenoid derivative extracted from the marine algae-derived endophytic fungus *Aspergillus wentii* EN-48. Zhang et al. (2013) used reverse docking to discover that this small molecule induces G2/M phase arrest and apoptosis of human hepatocellular carcinoma cells by co-targeting the Ras/Raf/MAPK proteins in their signaling pathways.

Here, we begin by introducing the basic principles of these three types of reverse screening methods, i.e., shape screening, pharmacophore screening and reverse docking, for the prediction of the protein targets of small molecules. Then, representative and classical software and online services for each method as well as the relevant databases are hierarchically categorized and systematically presented. Finally, we reviewed nearly all articles on the applications of these methods since 2000 and selected some typical examples to illustrate the use of these methods. By statistically analyzing these articles, we reveal the trends in the application of these three methods for computer-aided protein target prediction. In addition, we discuss their shortcomings and possible solutions as well as previous reviews of these reverse screening approaches for predicting the protein targets of small molecules.

## Methods

**Figure 2:**
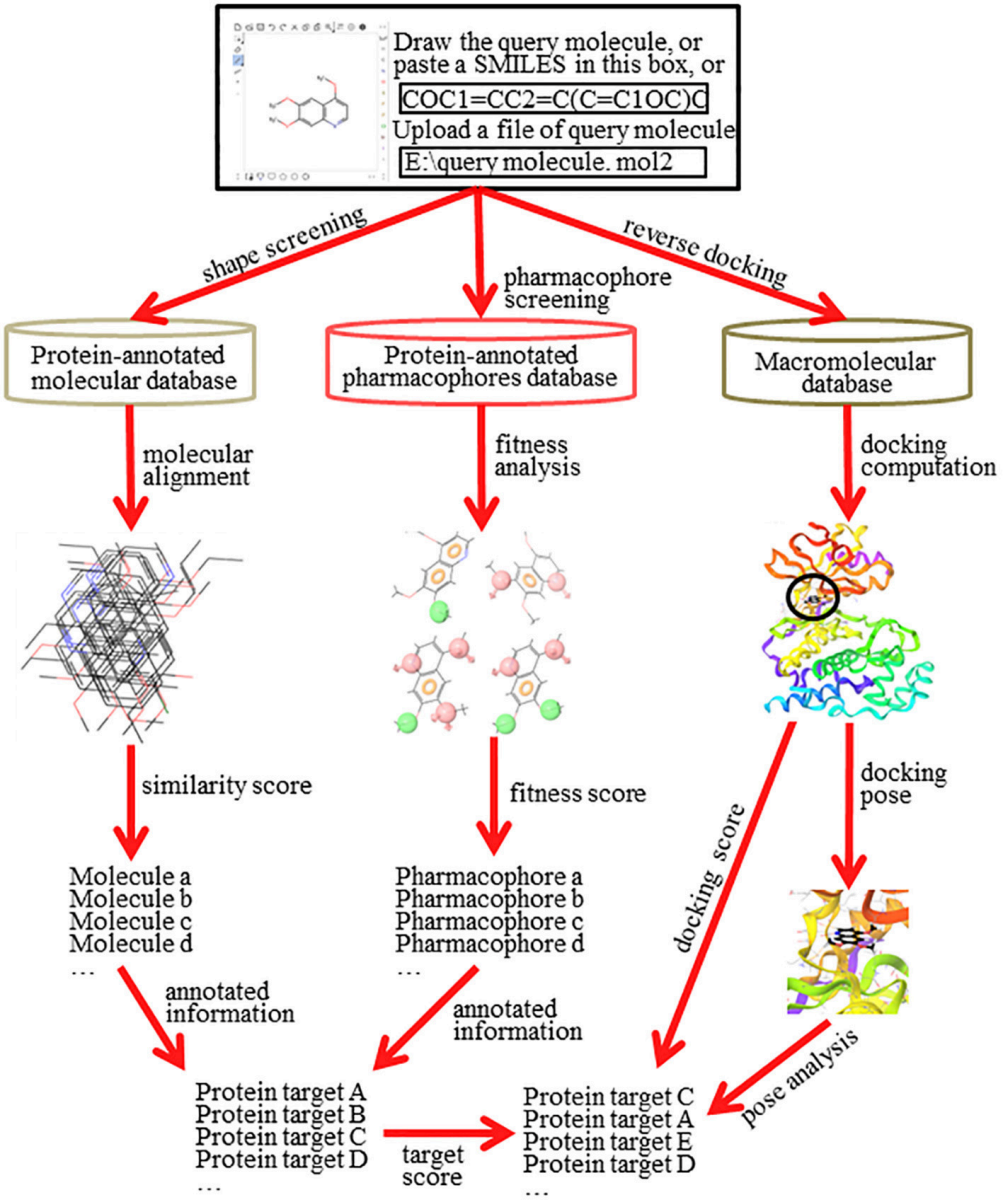
The principle and workflow of shape screening, pharmacophore screening, and reverse docking.

Reverse screening to search for unknown targets, unintended targets, or secondary targets of small-molecule drugs can be achieved by shape similarity screening, pharmacophore model screening, or reverse protein-ligand docking (Figure 2). These three different calculation approaches are complementary and can be used in conjunction with each other. By comparison, shape, and pharmacophore screening are simpler and faster, while reverse docking is more complex and slower. We will introduce these three methods in detail in the following sections.

### Shape screening

The basic principle of shape screening, from a two-dimensional (2D) perspective, is that structurally similar molecules may have similar bioactivity by targeting the same proteins. From a three-dimensional (3D) perspective, the basic principle is that molecules with similar volumes may have the potential to bind effectively to spaces of the same or similar size (considering the ligand-induced fit effect; Koshland, 1958) in the active pockets of proteins (Shang et al., 2017). To use shape screening to predict the targets of small molecules, a small-molecule ligand database annotated with protein targets is necessary. Then, the overall shape similarity of a query molecule to each ligand in the database can be measured individually. Finally, the protein targets for matched molecules with high similarity scores can be considered potential targets of the query molecule (Schuffenhauer et al., 2003). Shape screening involves two levels of mapping: the first mapping between the query molecule and the ligands in the database and the second mapping between the matched ligands in the database and their annotated protein targets (Figure 2).

Shape similarity comparison is based on the 2D or 3D topological structures of small molecules. Notably, 2D methods were originally created to obtain more of the same part between paired molecules, whereas 3D methods can be used to enhance scaffold diversity (Nettles et al., 2006). A universal descriptor for molecular similarity comparison in 2D methods is FingerPrint2D (FP2), which employs a simple bit vector to represent a variety of chemical characteristics and is encoded in a variety of software and databases (Bender et al., 2004). The most frequently used type of FP2 is extended-connectivity fingerprints (ECFPs), which are circular fingerprints. ECFPs symbolize circular atomic neighborhoods based on the Morgan algorithm and are designed especially for structural activity modeling (Rogers and Hahn, 2010). They have variable length: for example, ECFP4 refers to a diameter of 4 and ECFP6 to a diameter of 6 (Glem et al., 2006), both of which are encoded in TargetHunter (Wang L. et al., 2013). Molecular ACCess System (MACCS; Durant et al., 2002) is another commonly used FP2. It is a structure key-based fingerprint and is encoded in the 2D approach of the ChemMapper server (Gong et al., 2013). In addition to FP2, other descriptors are based on 2D topologies or paths, including the daylight fingerprint (http://www.daylight.com) encoded in ChemProt 3.0 (Kringelum et al., 2016) and the MDL structural key, another 2D descriptor (Durant et al., 2002). Structural matching based on 3D topology mainly compares the 3D geometries of the molecules, sometimes with the addition of pharmacophores (Lo et al., 2016), ElectroShapes (Armstrong et al., 2010), Spectrophores (Smusz et al., 2015), or other additional information. For example, WEGA (Yan et al., 2013) and gWEGA (Yan et al., 2014) compare only the volumes of two molecules, but SHAFTS (Lu et al., 2011), encoded in ChemMapper, incorporates pharmacophore matching when calculating the volume similarity.

The similarity of the descriptors in both 2D and 3D methods can be measured by the Tanimoto coefficient. The Tanimoto coefficient represents the ratio of the union to the intersection of the shapes of two molecules (Salim et al., 2003). For example, TargetHunter uses the Tanimoto coefficient to calculate the similarity among molecular fingerprints (Wang L. et al., 2013). The City-Block distance (CBD, also called the Manhattan or Hamming distance), which represents the difference between the sum of two molecular shapes and twice the overlap of two molecular shapes, can also be used to calculate the molecular similarity (Awale and Reymond, 2014). For example, SwissTargetPrediction uses this formula to calculate ElectroShape vectors in 3D comparisons (Gfeller et al., 2014).

Shape screening can be divided into two subclasses: indirect target prediction and direct target prediction. Indirect target prediction indicates that the potential targets of the query molecule are manually selected from the annotated protein targets of the matched database ligands. ROCS (Rush et al., 2005) and TargetHunter (Wang L. et al., 2013) are representative examples. These programs merely calculate the similarity scores between the query molecule and the matched ligands in the database but cannot reveal the complex relationships among the annotated protein targets of multiple matched ligands. In general, the annotated targets of any database ligand are not unique, and a protein target may also be annotated with multiple compounds (Rognan, 2010). Therefore, these programs can have high rates of false positives in target prediction and low accuracy in target searching.

Direct target prediction not only calculates the similarity score between the query molecules and the ligands in the database but also estimates the probability that the annotated targets of the matched ligands are targets of the query molecule. This extra process can reduce the false positive rate of target prediction and improve the accuracy of the target search. The probability that the annotated targets of the matched ligands are targets of the query molecule can be evaluated by multiple computational models or algorithms (the dotted line in Figure 2). For example, ChemMapper (Gong et al., 2013), which is based on a compound-protein network constructed from the top similar structures and their annotated targets, employs a random walk algorithm (Köhler et al., 2008) to calculate the probabilities of interaction between the query structure and the annotated targets of the hit compounds. In addition, SwissTargetPrediction (Gfeller et al., 2014) and CSNAP3D (Lo et al., 2016) use a cross-validation method and a network algorithm, respectively, to assess the probabilities that the annotated targets of the matched ligands are targets of the query molecule.

Because shape screening is based on the comparison of overall molecular shape, it may not be suitable for predicting the potential targets of molecules that are excessively large or small. Judging the potential targets of an oversized molecule is difficult because its best matched ligands usually show a low similarity score, and selecting the potential targets of an undersized molecule is difficult because its matched ligands are numerous with high similarity scores. Shape screening is suitable for predicting potential targets whose available inhibitors have sizes similar to that of the query molecule but is less fit for finding novel targets whose current inhibitors differ greatly in size from the query molecule but whose active pocket space is easily adjusted to bind diverse ligands due to a strong induced-fit effect.

### Pharmacophore screening

The basic principle of pharmacophore screening is that the binding of certain drugs with their protein targets is primarily determined by key functional pharmacophores (Rognan, 2010). Thus, the matching of these important pharmacophores can be used to search for new targets of small-molecule drugs (Fang and Wang, 2002). A pharmacophore is the spatial arrangement of functional characteristics that allows molecules to interact with target proteins in a particular binding mode, such as a hydrophobic center (H), hydrogen bond acceptor vector (HBA), hydrogen bond donor vector (HBD), positively charged center (P), or negatively charged center (N) (Kurogi and Güner, 2001). A pharmacophore model is the combination of pharmacophores in a pattern of ligand-protein interaction that give the final pharmacological effect (Leach et al., 2010). Similar to a ligand database for shape screening, a pharmacophore database also requires annotation with target protein information. In pharmacophore screening, the pharmacophore features of the query molecule are successively matched with the features of the pharmacophore models in the database. A higher matching degree indicates that the annotated protein target of the matched pharmacophore model has greater potential to be a target of the query molecule (Steindl et al., 2006). Pharmacophore screening also undergoes two levels of mapping: the first mapping is between the pharmacophore models of the query molecule and of the ligands in the database, and the second mapping is between the matched pharmacophore models of the ligands in the database and their annotated protein targets (Figure 2).

The pharmacophore database is built by pharmacophore modeling. The three construction methods are the use of ligands only, receptor structures only, or co-crystallized complex structures, which can be defined as ligand-based, structure-based and complex-based pharmacophore modeling, respectively. Ligand-based pharmacophore modeling was initially designed and is often used for traditional ligand-based virtual screening; an example is the quantitative structure–activity relationship (QSAR; Pulla et al., 2016). The most substantial common features shared by a group of active molecules can be easily extracted by using this method to form a good pharmacophore model to guide the further optimization of active compounds (Leach et al., 2010; Gaurav and Gautam, 2014). However, this approach is seldom used in reverse pharmacophore modeling due to the arbitrariness of pharmacophore models based on a single protein-annotated ligand.

The other two main methods, the use of only receptor structures and the use of protein-ligand complex structures, are forms of structure-based pharmacophore modeling (Gaurav and Gautam, 2014). In receptor-based methods, the pharmacophore features are first extracted from potential binding sites detected by specific protocols, and the pharmacophore models are then derived from the clustering of interaction point information and further refined or validated by using the input of the known ligands and their available or even calculated binding data (Chen and Lai, 2006). For instance, Pocket v.2 (Chen and Lai, 2006) and Catalyst SBP in Discovery Studio (DS) (BIOVIA, 2017) can both produce this type of pharmacophore database. In complex-based methods, pharmacophore models are simply generated via knowledge-based topological rules by using all features, such as hydrogen bonding information, charge, and hydrophobic contacts, based on the interactions between the co-crystallized ligands and receptor atoms (Sutter et al., 2011; Meslamani et al., 2012). Complex-based pharmacophore modeling is commonly used to construct pharmacophore databases, such as PharmaDB in Discovery Studio (Meslamani et al., 2012) and PharmTargetDB in PharmMapper (Liu et al., 2010), due to the stronger association between the built pharmacophore models and the experimentally verified ligand-protein interactions, which can improve the accuracy of target prediction.

The matching process between a pharmacophore model of the query molecule and the pharmacophore models in the pharmacophore database considers the alignment of two core components: pharmacophore feature types and the positions of the feature types (Wolber and Langer, 2005). The alignment of feature types is the matching between the pharmacophore features shared by the query molecule and the database ligands, such as matching between a hydrophobic feature in the molecular structure and those in database ligand pharmacophore models. The alignment of the feature positions is the pairwise matching of the distances between the fitted feature types in the pharmacophore models (Kabsch, 1976). For example, PharmMapper groups pharmacophores into triplets (e.g., H-H-H, H-HBA-HBD) and uses the vertexes of a triangle to represent the pharmacophore feature types and the side length of the triangle to measure the relative positions of these feature types (Liu et al., 2010).

In pharmacophore screening, the pairwise fitness score between pharmacophore models can be used directly as a basis for target evaluation. The fitness score includes the scores obtained from both the alignments between feature types and the alignments between the positions of each pair of pharmacophore models from the query molecule and database ligands. Higher fitness scores indicate higher probabilities (Wang X. et al., 2016). In addition, other matching information, such as the number of matched features and overall shape similarity, can also be used as additional references for target evaluation (Khedkar et al., 2007). If the pharmacophore scoring process does not consider the overall shape of the query molecules, it will be more likely to find pseudo protein targets with high fitness scores for a smaller query molecule because its limited pharmacophore features can be easily matched in the database (Wang X. et al., 2016). Thus, the target score must be recalculated to improve the prediction accuracy (the dotted line in Figure 2). For example, PharmMapper utilizes a normalized fitness score to re-rank the potential targets by standardizing a normal distribution of the fitness score to achieve a higher accuracy (Wang X. et al., 2017).

Since the construction of the pharmacophore database by structure-based pharmacophore modeling is not easy, the development of corresponding tools based on this principle has been somewhat limited. However, compared with shape screening, pharmacophore screening can improve the accuracy of prediction because it focuses on matching the key pharmacophore functional groups. In addition, it can ignore the total size of the molecule. As a result, pharmacophore screening can be used to search for potential targets of a query molecule with a large or small volume and can also be employed to find novel protein targets capable of binding a large diversity of ligands. Although PharmTargetDB, the PharmMapper in-house repository, does incorporate protein structural information, a pharmacophore database can be built to use ligands only. That is, constructing a pharmacophore database based on ligands with currently unavailable target structures is also useful for pharmacophore screening.

### Reverse docking

The basic principle of reverse docking is that the binding strength of a small-molecule ligand and a potential protein target is determined by their interaction energy (docking energy). To use reverse docking to predict the targets of a query molecule, a structure grid database of a large number of protein targets is normally required. Then, the query molecule is individually docked with each protein structure in the database. Each docking score is calculated. Finally, the protein targets are sorted according to their docking energy. Generally, a higher rank indicates a greater probability that the protein is a target of the query molecule. In contrast to shape screening and pharmacophore screening, reverse docking involves one level of mapping, which reflects the direct relationship between the query molecule and the target proteins (Figure 2). However, it is a complex process that includes recognition of a binding site, construction of the docking grid, a molecular docking algorithm, docking score calculation and target evaluation, among other steps (Lee et al., 2016).

In most cases, the active site of a protein is already known and can be determined from its co-crystallized small-molecule ligand. However, for some *apo*-form structures without co-crystallized ligands, the docking program must first recognize the active binding site of these proteins. If the *apo*-form structure is from a protein for which other co-crystallized structures are available, its active site can also be identified from those protein structures with co-crystallized ligands. Otherwise, *de novo* detection of the active site of the *apo*-form structure is required. The literature describes multiple ways to achieve this task. For example, Wang et al. (2012) uses the “divide-and-conquer” method in idTarget to search the surface structure of the entire protein and possible allosteric structures to find potential binding sites. Kuntz et al. (1982) describes a method that was later used in INVDOCK (Chen and Zhi, 2001) to define a binding site by a group of overlapping probe spheres of certain radii, which fill up a cavity and whose inward-facing surfaces cover the van der Waals surfaces of the protein atoms at the interface. Active site recognition is very useful in attempts to dock a query molecule into cavities other than the binding pockets of known ligands, which can increase the diversity of the binding between the query molecule and protein targets and improve the accuracy of reverse docking.

The database of protein targets used in reverse docking can be a library of protein crystal structure grids with recognized binding sites determined by co-crystallized ligands or available cavities. We can build these databases by continuously downloading a series of protein crystal structures from the Protein Data Bank (PDB); the time-consuming human-computer interaction processes (such as the deletion of water molecules, the addition of hydrogen atoms, and energy optimization) can be accomplished by using a molecular docking program, and the protein structure grids are finally generated. Traditional molecular docking programs, such as DOCK (Allen et al., 2015), AutoDock (Di Muzio et al., 2017), Schrödinger (Schrödinger, 2018) and Discovery Studio (BIOVIA, 2017), can be used to construct a custom target database for reverse docking to search for potential targets of a small molecule. Alternatively, the protein target database can also be a simply processed, automatically constructed protein structure database, and the grids can be generated after the programmed identification of active sites in the process of reverse docking; an example is the idTarget in-house database (Wang et al., 2012). Notably, the lack of a universal protein structure grid database and the need to build a new one for each docking program are the main reasons that reverse docking cannot be used as often as traditional structure-based virtual screening.

At present, reverse screening uses two main types of molecular docking techniques, originally developed in DOCK and AutoDock. DOCK (Ewing et al., 2001) adopts a “geometry matching method” to perform molecular docking by complementing the geometric shape of the docking ligands with that of the protein active site, usually including hydrogen binding sites and locally accessible sites (Shoichet et al., 2010). The matching process is performed by an “anchor and grow algorithm,” in which the anchor is a rigid portion of the ligand that is used to initialize a pruned conformation search, and grow refers to the generation of multiple conformations of the remaining segments to simulate the flexible docking of the ligand (Ewing et al., 2001). AutoDock uses a “docking simulation method” that employs the “genetic algorithm” to sample the conformations of a docking molecule inside a grid of the receptor binding pocket (Willett, 1995). In this algorithm, the molecule starts randomly at the receptor surface and undergoes orientation, translation and rotation to cause conformational changes until the ideal binding pose with the best binding energy is found (Morris et al., 2015). Among three reverse docking programs, INVDOCK (Chen and Zhi, 2001) and TarFisDock (Li et al., 2006) use the DOCK geometry matching method for molecular docking, while idTarget uses the AutoDock genetic algorithm for reverse docking (Wang et al., 2012).

Currently, almost all molecular docking programs can perform flexible-ligand docking due to the small size of the ligands; however, these programs still have difficulty in performing molecular docking with a fully flexible protein. Therefore, depending on the flexibility of the receptor proteins, reverse docking can also be classified into two types: rigid protein docking and semi-flexible protein docking. Although reverse docking with a rigid receptor is fast, it ignores ligand/receptor-induced fit effects. An example of a rigid protein docking program for reverse screening is TarFisDock (Li et al., 2006). Reverse docking with semi-flexible receptors can be achieved by various methods such as side-chain rotations (Liu H. et al., 2015), stretching of active pocket residues (Halgren et al., 2004), and ensemble docking (Lorber and Shoichet, 1998). For example, INVDOCK allows the amino acid residues of the receptor binding sites to rotate with the entry of the ligand, thereby simulating the ligand induced-fit conformational changes of receptors (Chen and Zhi, 2001). idTarget uses the docking of a query molecule into an ensemble of different receptor crystal structures after clustering (Wang et al., 2012) and thus simulates semi-flexible receptor docking by possible binding of the molecule with the distinct locations of the active pocket residues of the receptor in its different structures.

The docking score between a query molecule and receptors is an evaluation criterion for ranking its potential targets in reverse screening. Docking energy is a major method of scoring docking poses and normally refers to the interaction energy between the ligand and protein but may also include the energy of the ligand or the energies of both the ligand and the protein (or a part of the protein such as the binding pocket). For example, INVDOCK evaluates the docking structure by calculating the interaction energy between the ligand and receptor (Chen and Zhi, 2001), whereas idTarget scores the docking pose by calculating the energy of the ligand, the protein binding pocket and the interaction between them (Wang et al., 2012). According to the principle that the most stable structure has the lowest energy, a more negative docking energy results in stronger binding between the ligand and protein. The docking energy is calculated based on energy functions, which are mainly divided into three types: molecular mechanics energy functions, empirical energy functions, and semi-empirical energy functions. The molecular mechanics energy functions are more comprehensive and are rigorously defined by the sum of terms with clear physical meaning, including bond stretching, angle bending, torsion angles, van der Waals forces, electrostatic interactions, desolvation, or hydrophobic interactions, conformational entropy, and potentially others (Huang and Zou, 2010; Wang et al., 2011). In reality, the molecular mechanics energy functions used in the docking programs may include only some of these terms. For example, TarFisDock uses energy functions including only van der Waals and electrostatic interaction terms (Li et al., 2006). Empirical energy functions comprise weighted energy terms whose coefficients are obtained by reproducing the binding affinities of a benchmark data set of protein-ligand complexes (Gilson et al., 1997; Gilson and Zhou, 2007). For example, INVDOCK uses an empirical energy function based on simple contact terms, including hydrogen bond and non-bond terms, to calculate the ligand-protein interactive energy as the binding affinity (Chen and Zhi, 2001). Semi-empirical energy functions combine some molecular mechanics energy terms with empirical weights and/or empirical functional forms and have been widely used in computational docking methods (Raha and Merz, 2005). For example, idTarget follows the AutoDock 4 robust scoring functions (Huey et al., 2007) and employs a semi-empirical free energy function that includes hydrogen bonding, electrostatics, desolvation, and torsional entropy, whose weighting coefficients are derived from regression analysis of the experimental binding affinity information (Wang et al., 2011). In addition, reverse docking allows visual assessment of the docking poses by analyzing the number of hydrogen bonds, the presence or absence of critical hydrogen bonds and pi-pi conjugation, etc., as in traditional virtual screening, to further assist target evaluation for a more accurate prediction.

Reverse docking considers key elements of both shape screening and pharmacophore modeling. It determines whether or not the size of a query molecule can fit inside the binding pocket of a protein target by docking and scores the interaction of the key pharmacophore groups in the molecule and the targets to perform target evaluation. Thus, reverse docking could be the most comprehensive of the three methods in principle. However, similar to traditional molecular docking, it also has the following shortcomings: incompleteness of the search space, inaccuracy of the scoring function, and extensive calculation (Lee et al., 2016). Relative to traditional docking, reverse docking has the additional problem that the sizes of the active pockets of proteins defined by co-crystallized ligands are inconsistent. Even if the docking pockets can be defined as being a universally equal size, the residue density of different protein binding pockets may vary, resulting in differences in the calculation ranges for the binding interaction energies. Therefore, reverse docking suffers from a rationality problem, as it is unable to normalize binding energies for the correct sorting of potential targets. Nevertheless, reverse docking can serve as an effective method to complement shape and pharmacophore screening when the protein structures are available.

### Software and online services

**Figure 3:**
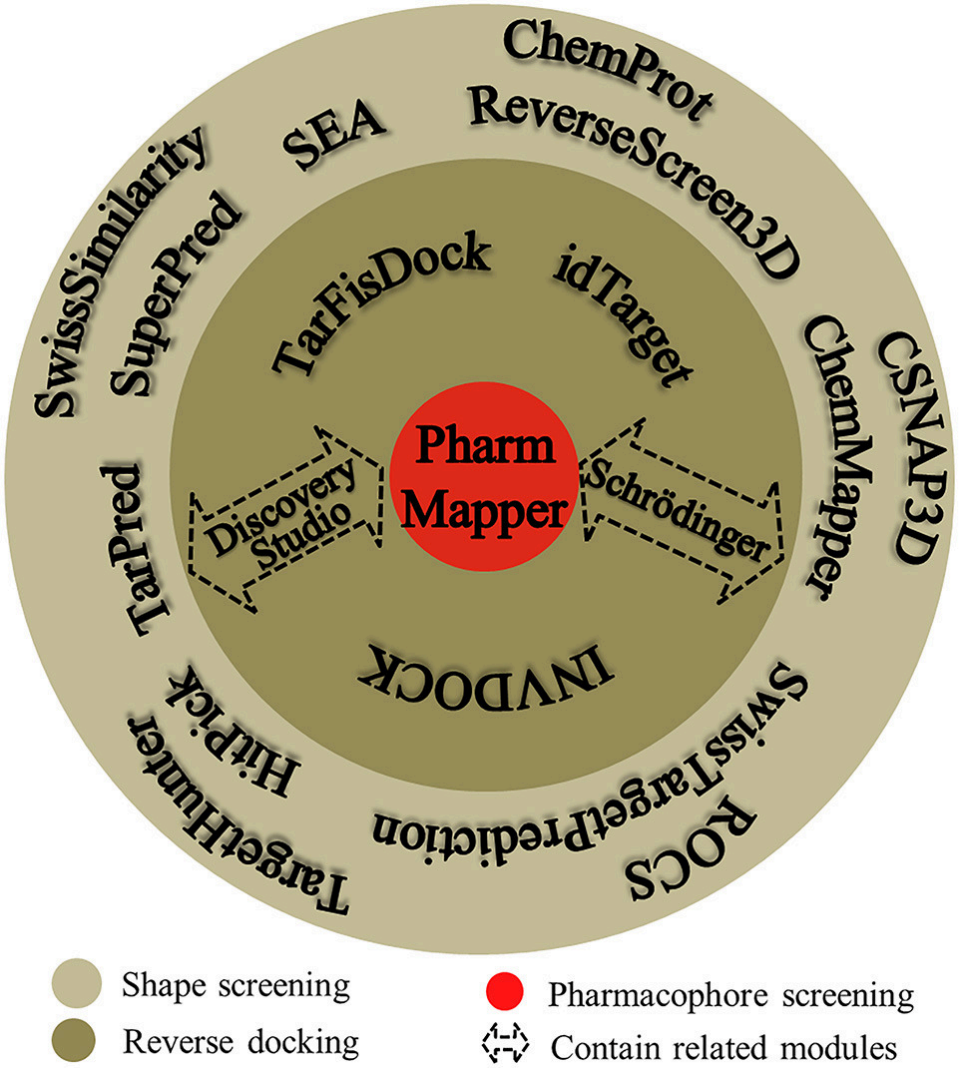
Software and online services for shape screening, pharmacophore screening, and reverse docking.

Many software programs, some of which are available as online services, can be used for reverse screening to predict protein targets of small molecules, but the numbers of online tools available for the three methods are quite different. Shape screening tools are the most numerous and include more than a dozen, such as ChemProt (Kringelum et al., 2016), ROCS (Rush et al., 2005), ChemMapper (Gong et al., 2013), and the SEA search server (Keiser et al., 2007). They are listed in the outer ring of Figure 3. By contrast, the only tool available for pharmacophore screening is PharmMapper (Liu et al., 2010), as shown in the inner ring of Figure 3. The main tools available for target searching by reverse docking are TarFisDock (Li et al., 2006), idTarget (Wang et al., 2012) and INVDOCK (Chen and Zhi, 2001), which are illustrated in the middle ring of Figure 3. A few large software packages, such as Schrödinger and Discovery Studio, also contain related modules that perform reverse screening, but they can be used only for the indirect prediction of potential targets of small molecules. These tools require users to build their own databases or perform other relevant processing steps. We have summarized the basic information on these tools, organized according to their characteristics, in Table 1. In addition, for each type of software and online service, we have provided more detailed descriptions of a few classic representatives.

**Table 1:** Characteristics of reverse screening tools.

**Name**	**Required format**	**Search method**	**Coverage**	**Reference website**	**First version**	**Last version**	**State**
SuperPred	SMILES, Pubchem-Name	2D similarity	341,000 compounds, 1800 targets, 665,000 compound-target interactions	http://prediction.charite.de/	2014	2014	Accessible
HitPick	SMILES	1NN similarity searching and Laplacian-modified naïve Bayesian target models	145,549 chemical-protein interactions collected from STITCH 3.1	http://mips.helmholtz-muenchen.de/hitpick/cgi-bin/index.cgi?content=targetPrediction.html	2013	2013	Accessible
ChemMapper	SMILES, MOL2, SDF, SMI	SHAFTS, USR, FP2, MACCS, and random walk algorithm	Nearly 300,000 chemical structures and >3 million compounds	http://lilab.ecust.edu.cn/chemmapper/	2013	2016	Accessible
SEA server	SMILES	FP2 and BLAST-like model	Hundreds of target-ligand sets	http://sea.bkslab.org/	2007	2007	Accessible
ReverseScreen3D	SMILES	Hybrid 2D&3D	Automatically updated from RCSB PDB	http://www.modeling.leeds.ac.uk/ReverseScreen3D	2011	2011	Inaccessible
TarPred	SMILES	KNN-based data fusion with molecular similarity	533 individual targets with 179,807 active ligands	http://202.127.19.75:5555/	2015	2015	Inaccessible
SwissTargetPrediction	SMILES	Five species, FP2, 3D similarity	280,000 compounds and >2000 targets	http://www.swisstargetprediction.ch/index.php	2014	2014	Accessible
SwissSimilarity	SMILES	FP2, five 3D methods	>30 chemical databases covering drugs, bioactive compounds, etc.	http://www.swisssimilarity.ch/	2016	2016	Accessible
ChemProt	SMILES, name	MACCS, FP2, daylight-like fingerprints	>1.7 million unique chemicals and >20,000 proteins	http://potentia.cbs.dtu.dk/ChemProt/	2011	2016	Accessible
TargetHunter	SMILES	ECFP6, ECFP4, FP2	CHEMBL Version 22	http://www.cbligand.org/TargetHunter/	2013	2016	accessible
CSNAP3D	SMILES, SDF	3D similarity, network algorithms	Based on CHEMBL database	http://services.mbi.ucla.edu/CSNAP/index.html	2016	2016	accessible
ROCS	SDF, MOL2, PDB	3D similarity	User prepared	https://www.eyesopen.com/rocs	2006	2017	Accessible
PharmMapper	MOL2, SDF	Pharmacophores	23,236 proteins covering >53,000 pharmacophore models	http://lilab.ecust.edu.cn/pharmmapper/	2010	2017	Accessible
TarFisDock	MOL2	DOCK4.0	Based on the PDTD, which contains 1207 entries covering 841 known and potential drug targets	http://www.dddc.ac.cn/tarfisdock/	2006	2008	Inaccessible
idTarget	PDB, MOL2, pdbqt, cif	MeDock, divide-and-conquer	All protein structures in the PDB	http://idtarget.rcas.sinica.edu.tw	2012	2012	Accessible
INVDOCK	NA	DOCK	An in-house database (9000 protein and nucleic acid entries)	http://bidd.nus.edu.sg/group/softwares/invdock.htm	2001	NA	Accessible
Discovery Studio	SDF, MOL2, PDB	Pharmacophores	140,000 receptor-ligand pharmacophore models.	http://accelrys.com/products/collaborative-science/biovia-discovery-studio/pharmacophore-and-ligand-based-design.html	2012	2017	Accessible

### Shape screening

At present, many online services are available to search for targets of small-molecule drugs by shape screening. According to whether these services and software programs can directly sort the potential protein targets by probability or not, we classified them into direct target prediction tools, such as SuperPred (Dunkel et al., 2008), HitPick (Liu et al., 2013), ChemMapper, SEA search server, ReverseScreen3D (Kinnings and Jackson, 2011), TarPred (Liu et al., 2015a), and SwissTargetPrediction, and indirect target prediction tools, such as SwissSimilarity (Zoete et al., 2016), ChemProt, TargetHunter (Wang L. et al., 2013), CSNAP3D (Lo et al., 2016), and ROCS. These categories, respectively, are located on the inside and outside of the outer ring in Figure 3. A brief introduction to these services, including their input and output formats, shape similarity calculation methods, database information and website links, is provided in Table 1. Because indirect target prediction services require the manual selection of protein targets, we do not provide a more detailed overview of these tools here. We chose the SEA search server among the direct target prediction services as a representative for further description because it is the oldest and most widely used shape-screening service (Keiser et al., 2007).

As a web-based target prediction tool, SEA was developed in 2007, and it performs quantitative classification and target association based on the chemical similarity of protein-related ligands (Keiser et al., 2007). SEA supports only the SMILES format for the input of query molecules for target prediction. After receiving the relevant information about the query compound, SEA performs a pairwise comparison of a 2D similarity metric in a collection of ~65,000 ligands annotated with drug targets, in which most annotations contain hundreds of ligands (Keiser et al., 2007). SEA then clusters the ligands based on their chemical similarity into hundreds of sets, relating their corresponding annotated targets to each other quantitatively, and further uses a model resembling that of BLAST (Mount, 2007) to link these sets together in a minimal spanning tree (Keiser et al., 2007). Next, a statistical model is used to rank the significance (*E*-value) of the resulting similarity scores of each set in the minimum spanning tree. Finally, SEA produces a list of Max Tanimoto coefficients (MaxTc) and *E*-values. A larger similarity score (maxTC) with smaller significance score (*E*-value) indicate a higher rank, and there is a greater probability that the protein is a potential target.

### Pharmacophore screening

PharmMapper, the only web server to screen the potential protein targets of a query molecule based on pharmacophore modeling (Figure 3), was developed in 2010 (Liu et al., 2010; Wang X. et al., 2017). PharmMapper uses a triangle hashing mapping method to match the pharmacophore models between the compound and the internal database ligands to predict potential protein targets of a query molecule (Liu et al., 2010). A brief introduction to this online tool is also given in Table 1, including the input and output formats, database information and website link. Its in-house database, PharmTargetDB, will be described in detail in the Databases section. PharmMapper supports the Tripos Mol2, MDL and SDF formats for the input of a 2D or 3D query molecule structure to begin a job. Next, PharmMapper flexibly aligns the molecule with each protein pharmacophore model in its database and calculates the fit score between the query molecule and the pharmacophore models (Liu et al., 2010). Subsequently, PharmMapper ranks candidate targets according to the fit score (Liu et al., 2010) or according to normalized fit scores standardized by using a two-dimensional Z-transformation algorithm on the ligand and pharmacophore target dimensions (Wang X. et al., 2016), and records the aligned pharmacophore pose for the query molecule and targets. With the default setting, the top 300 target hits of the prioritizing list are outputted, and users can select candidate proteins based on both these fit scores and the aligned pose for further bioassay experiments (Liu et al., 2010; Wang X. et al., 2017).

### Reverse docking

TarFisDock, idTarget, and INVDOCK (Figure 3) are three reverse docking programs that are currently widely used in predicting the targets and mechanisms of various active biomolecules. A brief introduction to these tools is given in Table 1, including the input and output formats, database information and website links. Here, we will also provide a slightly more detailed description of these three tools.

TarFisDock, a web-based tool for predicting the potential binding targets of a given ligand, was first released in 2006 (Li et al., 2006) and last updated in 2008 (Gao et al., 2008). TarFisDock uses reverse docking to search for all possible protein binding partners of small molecules from a potential drug target database called PDTD (Li et al., 2006), which will be described further in the Databases section. This program supports only the mol2 format for the input of the query molecule. TarFisDock (Li et al., 2006) uses the docking program DOCK 4.0 to perform molecular docking between the given molecule and each protein in the PDTD, and it calculates their binding energy based on van der Waals and electrostatic interaction terms by using the Amber force field (Weiner et al., 1984). TarFisDock can output a list of the top 2, 5, or 10% target hits according to binding energy. The main limitation of TarFisDock is the insufficient number of target proteins in the PDTD. The PDTD initially released in 2006 contained only 698 protein structures (Li et al., 2006) and was expanded to contain >830 protein targets in 2008 (Gao et al., 2008). TarFisDock considers the flexibility of the small molecules but has yet to consider the flexibility of the protein targets.

In 2012, the web server idTarget (Wang et al., 2012) was developed to predict the potential binding targets of small molecules via a divide-and-conquer reverse docking approach. To improve the efficiency of target prediction, idTarget uses a contraction-and-expansion strategy to differentiate the protein structures at different levels for molecular docking into the families of homologous structures by clustering almost all of the protein structures deposited in the PDB (Wang et al., 2012). idTarget supports multiple coordinate formats, including pdbqt and mol2, for the input of the given molecule. Then, the program uses MEDock (Chang et al., 2005) to initially generate a large number of conformations of the query molecule and directly orient them inside the grid box of the binding site for molecular docking (Wang et al., 2012). Subsequently, idTarget assesses the binding pose by using semi-empirical score functions derived from quantum chemical charge models and robust regression analysis (Wang et al., 2012). Finally, the program outputs two sets of results, both of which are ranked in ascending order of the predicted binding free energy (ΔG^Pred^). One set of results is listed according to the names of the proteins, while the other is listed according to the names of the homologous families (Wang et al., 2012). In addition, idTarget provides two modes for searching binding poses, scanning mode and fast mode (Wang et al., 2012). In “scanning mode,” molecular docking is performed individually for each protein structure in the database. In “fast mode,” the ligand is docked simultaneously to the binding sites of the superposed homologous protein structures, and the binding poses are further minimized by adaptive local sampling (Shindyalov and Bourne, 1998). The fast mode performs quick searches via docking between the ligand and the common binding sites after the protein structures of each homologous family are pre-aligned, but the scanning mode does not limit the docking conformation searches to these predetermined binding sites (Wang et al., 2012). Both modes uses the strategy of ensemble docking (Lorber and Shoichet, 1998) to consider the flexibility of the receptor indirectly (Wang et al., 2012).

INVDOCK (Chen and Zhi, 2001), an online service for ligand-protein reverse docking that runs on both Windows and Unix platforms, was developed in 2001. INVDOCK has an in-house protein target database of 9000 protein and nucleic acid entries (Chen and Zhi, 2001). It supports standard 3D ligand structure files, such as the SDF and MOL formats. INVDOCK sets cavities on the protein surface that are covered by a large portion of spherical probes as active binding pockets. The automatic docking is performed by multi-configuration shape matching between the molecule and cavities. Then, torsion optimization and energy minimization are performed on the molecule and on the protein residues in the binding region (Chen and Zhi, 2001). Finally, the simplified DOCK scoring method is used to score the binding energy, and the protein targets are ranked in ascending order by the ligand-protein interaction energy function (ΔE_LP_; Chen and Zhi, 2001). INVDOCK also considers the flexibility of the protein via a limited torsion space sampling of rotatable bonds in the side chains of the target residues at the binding site (Chen and Zhi, 2001).

### Integrated software suites

Some drug design software suites, such as Schrödinger (2018) and Discovery Studio (BIOVIA, 2017), can also be used in shape screening, pharmacophore screening, and reverse docking to predict the protein targets of small molecules.

The Schrödinger modules for reverse screening are “Shape Screening,” “Pharmacophore Modeling,” and “Docking”. However, Schrödinger does not provide any ligand or protein database that can be used for reverse screening, and thus, users must provide the databases themselves. “Shape Screening” and “Pharmacophore Modeling” require a protein-annotated ligand database that can be generated by a simple process from the ligand library Ligand Expo, which can be downloaded from the PDB database (Feng et al., 2004; Rose et al., 2015). Each small-molecule ligand in this library is annotated with its co-crystallized protein target information. The protein structure grid database for reverse docking can be constructed by users via grid generation by the “Docking” module after the “Protein Presentation Wizard” is used to pre-process the protein crystal structure coordinates from the PDB, such as to remove water molecules and add hydrogens. Then, reverse docking can be performed by using “Glide Cross Docking” to dock a given molecule with multiple proteins simultaneously. Although the number of proteins for simultaneous docking in “Glide Cross Docking” is limited (normally ~ 50), users can write their own scripts to run reverse docking for one ligand and many proteins. We have performed several reverse screening tasks by using these modules in the Schrödinger software package (Kim et al., 2014; Lim et al., 2014; Wang Z. et al., 2016).

The “Pharmacophore” and “Receptor-Ligand Interaction” modules of Discovery Studio can be used for reverse screening. Users can select shape screening or pharmacophore screening by using the “Ligand Profiler” tool in the “Pharmacophore” module. This tool allows users to upload a database or use the Ligand Profiler Pharmacophore Database, PharmaDB, provided in the software. This database is generated from an annotated database of druggable binding sites called scPDB (Desaphy et al., 2015) based on the PDB and contains the molecular structure and corresponding pharmacophores for calculation of the shape and pharmacophore similarity. The protein crystal structure database for reverse docking must be prepared by the user. These crystal structures can be defined by the “Define and Edit Binding Site” in the “Tools” menu of the “Receptor-Ligand Interaction” module. “Libdock Batch Mode” in the “Protocols” menu of the “Receptor-Ligand Interaction” module can be used for batch docking and has the same effect as reverse docking. Because of the different algorithms and databases needed, the advantages and disadvantages of these two software suites for reverse screening have yet to be evaluated.

### Databases

**Figure 4:**
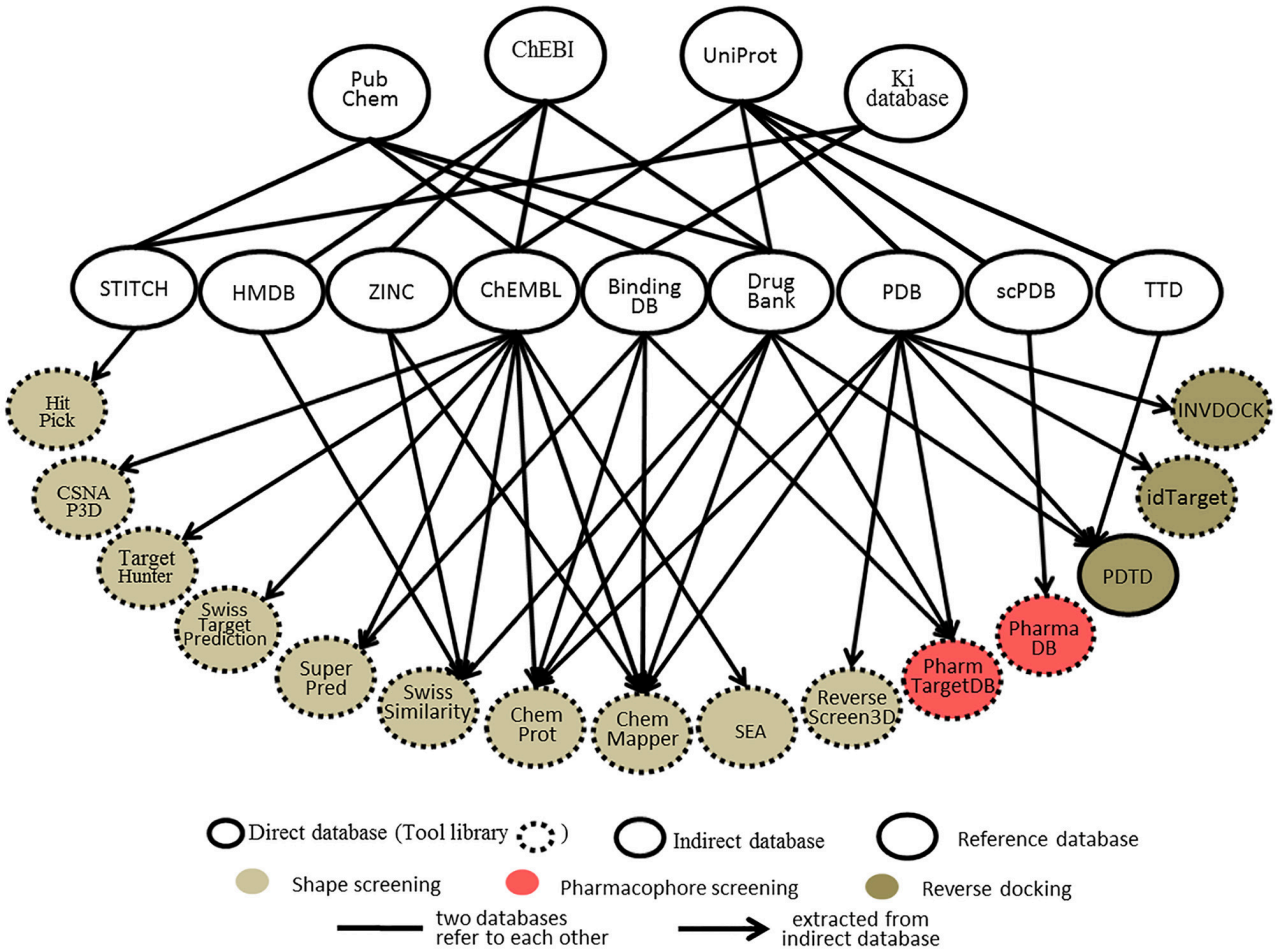
The relationships among direct databases, indirect databases, and reference databases used in reverse screening.

Databases, whether protein-annotated ligand structure/pharmacophore databases or protein structure grid databases, are key elements of reverse screening. Although reverse screening has been under development for nearly two decades, no general or benchmarked database is available for use in different methods or programs. Here, we have classified the existing relevant databases at different levels (Figure 4). The first class of databases is associated with software built by software developers and used for program running. We call these databases “software databases” or “direct databases” (shown at the bottom layer of Figure 4). Each database in this class is named for its corresponding software or referred to as the software in-house database. The second class of databases provides resources describing annotated ligands or target structures, but the users must process and collect these resources to construct direct databases for reverse screening. We call these databases “indirect databases” (shown in the middle layer of Figure 4), and examples include the PDB (Rose et al., 2015) and ZINC (Sterling and Irwin, 2015). The third class of databases can provide a large amount of information about ligands or proteins that can be used for reverse screening, but collecting and organizing the information from these databases to construct direct databases is difficult. However, we can use them to search for various information resources, such as additional targets, the bioactivities of matched molecules, or the signaling pathways of potential target proteins for further analysis of the reverse screening results. We call these databases “reference databases” (shown in the upper layer of Figure 4); examples include PubChem (Kim et al., 2016) and UniProt (Pundir et al., 2015). Relevant information on several direct databases used for reverse screening can be found in Table 1. In addition, information on indirect and reference databases, including the database coverage and website links, is listed in Table 2. In the following paragraphs, we provide a slightly more detailed introduction to these three classes of databases and their relationships with each other.

**Table 2:** Characteristics of indirect and reference databases.

**Database name**	**Aims**	**Coverage**	**Website**	**Last update**
**INDIRECT DATABASE**				
STITCH	A database of known and predicted interactions between chemicals and proteins	9.6 million proteins, 0.5 million chemicals	http://stitch.embl.de/	2017
HMDB	A comprehensive, high-quality, freely accessible online database of small-molecule metabolites found in the human body	74,507 metabolite entries, 5,701 protein sequences linked to metabolite entries	http://www.hmdb.ca/	2017
ZINC	Database of commercially available compounds for virtual screening	>100 million purchasable compounds	http://zinc15.docking.org/	2015
ChEMBL	An open large-scale bioactivity database	2,101,843 compound records, 1,735,442 distinct compounds	https://www.ebi.ac.uk/chembl/	2017
BindingDB	Database of measured binding affinities	2037 FDA-approved small-molecule drugs, 241 FDA-approved biotech (protein/peptide) drugs, 96 nutraceuticals, >6000 experimental drugs	https://www.bindingdb.org/bind/index.jsp	2011
DrugBank	Database combining detailed drug data with comprehensive drug target information	8,261 drug entries, 4,338 non-redundant protein sequences	https://www.drugbank.ca/	2017
PDB	Crystal structures of macromolecules and ligands	133,093 macromolecular structures, 53,025 citations, 23,711 ligands	http://www.rcsb.org/pdb/home/home.do	2017
scPDB	Druggable binding sites from the PDB	9283 entries, 3678 proteins, 5608 ligands	http://bioinfo-pharma.u-strasbg.fr/scPDB/	2013
TTD	Information on therapeutic protein and nucleic acid targets, relevant diseases, pathway information and the corresponding drugs	2,589 targets, 31,614 drugs, 20,278 small molecules, 653 antisense drugs	http://bidd.nus.edu.sg/group/cjttd/	2011
**REFERENCE DATABASE**				
PubChem	Information on chemical substances and their biological activities	>235 million substance descriptions, 90 million unique chemical structures, 230 million bioactivity outcomes from over one million biological assay experiments	https://pubchem.ncbi.nlm.nih.gov/	2017
CHEBI	A database and ontology of molecular entities focused on “small” chemical compounds	>52,450 compounds	http://www.ebi.ac.uk/chebi/	2017
UniProt	Resource for protein sequences and functional information	Swiss-Prot (555,100), TrEMBL (88,032,926)	http://www.uniprot.org/	2017
PDSP Ki database	A unique resource in the public domain that provides information on the abilities of drugs to interact with an expanding number of molecular targets	60,231 annotated ligands	https://kidbdev.med.unc.edu/databases/kidb.php	2017

### Direct databases

#### Direct databases used in shape screening

Each online service for shape screening has its own in-house database, except ROCS (Rush et al., 2005), which requires users to prepare their own protein-annotated ligand databases (Mori et al., 2015). The database information for 12 shape-screening software programs is shown in the “coverage” column of Table 1. Among the 11 direct databases, TargetHunter, CSNAP3D and ReverseScreen3D do not give specific capacity data; for the former two, the information is not published, and the latter is noted only as updated with updates to the RCSB PDB, according to the literature (Kinnings and Jackson, 2011).

Figure 4 shows 10 direct databases with clear sources, including HitPick, CSNAP3D, TargetHunter, SwissTargetPrediction, SuperPred, SwissSimilarity, ChemProt, ChemMapper, SEA, and ReverseScreen3D. These databases are basically constructed by extracting data from indirect databases (Figure 4). For example, ChemMapper is built from several public databases, including ChEMBL (Gaulton et al., 2017), DrugBank (Law et al., 2014), BindingDB (Gilson et al., 2016), KEGG (http://www.kegg.jp/kegg/) and the PDB. It collects bioactive targets and pharmacological information on small molecules, each of which has various pre-generated conformations for 3D similarity screening (Gong et al., 2013). ChemProt 3.0 (Kringelum et al., 2016) includes all chemical-protein interaction data from the available open source databases, including ChEMBL (version 19), BindingDB, the Psychoactive Drug Screening Program (PDSP) Ki database (Roth et al., 2000), and DrugBank, as well as clinical information from the Anatomical Therapeutic Chemical (ATC) Classification System (Wang Y. C. et al., 2013) and side effect data from Sider (Kuhn et al., 2016). The SwissSimilarity database gathers protein-annotated ligands mainly from four indirect databases, namely, HMDB (Wishart et al., 2009), ZINC, ChEMBL, and DrugBank, as well as from some reference databases, such as Chemical Entries of Biological Interest (ChEBI; Hastings et al., 2016). The SuperPred (Nickel et al., 2014) database consists of a large data set of ligand-target interactions from two indirect databases, ChEMBL and BindingDB. The CSNAP3D (Lo et al., 2016), Target Hunter (Wang L. et al., 2013), SwissTargetPrediction (Gfeller et al., 2014), and SEA (Keiser et al., 2007) databases are all constructed by taking ligands with protein target information from ChEMBL. The HitPick (Liu et al., 2013) database collects information from the STITCH database (Szklarczyk et al., 2015), and the information in ReverseScreen3D (Kinnings and Jackson, 2011) is extracted from the PDB database. In addition, the TarPred (Liu et al., 2015a) database, which is not shown in Figure 4, is a compound-target-disease database built by gathering information from the Comparative Toxicogenomics Database (CTD; Davis et al., 2017) and UniProt.

#### Direct databases used in pharmacophore screening

Two direct databases used for pharmacophore screening are shown in Figure 4. PharmTargetDB is the in-house database of the PharmMapper server (Liu et al., 2010), and PharmaDB (Meslamani et al., 2012) is the direct database deposited and used in Discovery Studio. Since these two databases are updated when the software updates, the different versions of PharmTargetDB and PharmaDB have different data capacities. The numbers of pharmacophore models in the newest versions are also shown in Table 1.

The pharmacophore models in PharmTargetDB are derived from the DrugBank, BindingDB, PDB, and PDTD databases. These models are built by extracting pharmacophore features within cavities by using the receptor-based pharmacophore modeling program Pocket 2.0 (Chen and Lai, 2006) after the binding sites of given protein structures are detected and ranked based on their druggability scores by using the software CAVITY (Yuan et al., 2013) for binding site detection (Wang X. et al., 2017). The original version of PharmTargetDB contained more than 7,000 pharmacophore models built from co-crystallized complex structures of protein targets (Liu et al., 2010). A new version of PharmMapper was published in 2017 (Wang X. et al., 2017), and the new PharmTargetDB is six times larger than the previous one, with a total of 23,236 proteins covering 51,431 pharmacophore models. PharmaDB is the pharmacodynamics database for Discovery Studio drug design software, and its pharmacophore models are constructed by using the Receptor-Ligand Pharmacophore Generation Protocol with default settings based on the binding information of ligand and protein complexes in the scPDB database. The original version of PharmaDB contained 68,056 pharmacophore models annotated with receptor information (Meslamani et al., 2012). The latest version of PharmaDB includes 140,000 pharmacophore models (BIOVIA, 2017), and users can utilize it in Discovery Studio to perform rapid reverse pharmacophore screening to search for protein targets of small molecules.

#### Direct databases used in reverse docking

The direct databases used in reverse docking are collections of target structure grids, which are usually generated from protein crystal structures by using docking programs or their auxiliary software tools. Before grid generation, the target crystal structures downloaded from the PDB must be preprocessed to remove ions and waters, add hydrogens and define the binding pocket. The original 3D protein structures can also be downloaded from some PDB derivative databases, such as the PDBbind-CN Database (Liu Z. et al., 2015), where all valid ligand-protein structures in the PDB are identified and collected.

PDTD, INVDOCK, and idTarget are the three direct databases used for reverse docking shown in Figure 4. Among them, PDTD is the only open database, and it can be downloaded as a compressed file, which is then decompressed as a collection of two types of structure files. The first type is the preprocessed protein structure file in the PDB and mol2 formats, and the second type is the active site structure file in PDB format. These two types of structure files for any PDB entry can be downloaded independently and viewed using the molecular visualization tool plug-in (Gao et al., 2008). Currently, this database contains more than 1,100 protein entries with 3D structures in the PDB and covers 841 diverse drug targets associated with diseases, biological functions, and signaling pathways (Gao et al., 2008). The binding (active) sites of these protein structures were defined by a data set of amino acid residues within 6.5 Å of the bound ligand (Gao et al., 2008). The INVDOCK and idTarget databases are not published but are the in-house databases of the corresponding programs. In addition, in contrast to the binding sites defined by the co-crystallized ligands in the PDTD, the binding sites in the INVDOCK and idTarget databases are generated from the available cavities by a search performed with spherical probes. The protein structures in these two databases also originate from the PDB. The INVDOCK in-house database, constructed in 2001, collects 9000 proteins and nucleic acid structures from the PDB database (Chen and Zhi, 2001). Each receptor structure grid in this database is constructed by first calculating the inward-facing surface covering the interface of van der Waals surfaces of the receptor crystal structure with a probe sphere 1.4 Å in radius. The binding site is then defined as the surrounding space within 15 Å of the center of the cavity formed by the combination of neighboring spheres covered by protein atoms in more than 50% of directions. Finally, grid generation is performed (Chen and Zhi, 2001). The idTarget database collects all protein structures in the PDB and is regularly updated when the PDB updates (Wang et al., 2012). The binding sites of each protein in the database are dynamically determined, and the grids are constructed by the “divide-and-conquer” method according to the size of the query molecule (Wang et al., 2012). Theoretically, idTarget could be the most extensive and complete database among the three for reverse docking.

#### Indirect databases

Indirect databases are rich in ligand and target information and can be simply processed to build direct databases for reverse screening. Nine indirect databases are shown in the middle layer of Figure 4, and a brief introduction to these databases, including the coverage, update time and website link, is given in Table 2. Among these nine indirect databases, ZINC, ChEMBL, BindingDB, and DrugBank mainly include structural information on ligands and their target annotations, whereas the PDB, scPDB, and Therapeutic Target Database (TTD) mainly provide 3D structures of proteins with ligand binding information. The 15 direct and in-house software-associated databases used for reverse screening are essentially extracted or constructed from these indirect databases. These indirect databases are also extensively linked to reference databases. For example, DrugBank and ChEMBL have mutual data exchange with PubChem, ChEBI, and UniProt. BindingDB (Gilson et al., 2016) collects data from the PubChem and PDSP Ki databases. The scPDB and PDB share protein information with UniProt, including sequences and crystal structures. In addition, HMDB (Wishart et al., 2007) and ZINC share compound information with ChEBI, and the TTD database collects its information on therapeutic protein targets from UniProt.

Users can employ indirect databases to build their own databases when they use large commercial software suites for molecular drug design, such as Schrödinger and Discovery Studio, to perform reverse screening. For example, users can collect small molecules with explicit target annotation from the DrugBank, ChEMBL, and ZINC databases to construct a ligand database for shape screening. They can also use ligand-protein binding information from the BindingDB, scPDB, and PDB databases to build a pharmacophore model database for pharmacophore screening or a protein structure grid database for reverse docking. Notably, Ligand Expo (Feng et al., 2004) from the PDB can be easily used to build in-house program databases for shape or pharmacophore screening after non-ligand small molecules, including metal ions and solvent molecules, are removed. In addition, a collection of protein crystal structures downloaded from the PDB can be used to generate a protein structure grid database for reverse screening. In fact, we built our own databases for use in Schrödinger based on this Ligand Expo database and the protein structures in the PDB, and we then performed shape screening and reverse docking to search for the protein targets of several natural compounds (Kim et al., 2014; Lim et al., 2014; Wang Z. et al., 2016).

#### Reference databases

Reference databases normally contain a very large amount of information on small-molecule compounds and proteins. However, extracting the rich information resources from these databases to establish direct databases for reverse screening can be difficult for users. Nevertheless, we can utilize these databases to search for additional information on the matched molecules and their potential protein targets from reverse screening results.

The four main reference databases, PubChem, ChEBI, PDSP Ki, and UniProt, which are closely associated with the nine indirect databases, are shown in the upper layer of Figure 4. A brief introduction to these four databases is also provided in Table 2. PubChem consists of three interrelated sub-databases: substances, compounds, and bioassays. The first two sub-databases provide information on the chemical structure and other properties of small molecules, and the sub-database of bioassays gives information on their pharmacological properties and biological targets (Kim et al., 2016). ChEBI is a freely available dictionary of molecular entities focused on “small” chemical compounds. ChEBI and ChEMBL are both sites of the European Molecular Biology Laboratories, and their data occasionally overlap. Compared with ChEMBL, ChEBI is more focused on “molecular entity” information, such as the chemical and biological roles and applications of a small molecule, rather than on biological target information (Hastings et al., 2013, 2016). The PDSP Ki database is a unique open resource that provides information on the ability of drugs to interact with increasing numbers of molecular targets. The key data in this database are the Ki activity data on ligands internally derived or from published articles, but it also includes information on protein-annotated ligand structures, protein-ligand affinities, and article sources (Roth et al., 2000). UniProt (Pundir et al., 2015) is a freely accessible resource of protein sequence and function information extracted from gene sequencing and published literature that undergoes quality assurance by curator-evaluated computational analysis (Poux et al., 2017).

The PubChem, ChEBI, UniProt, and Ki databases are the four major reference databases used by researchers. However, more reference databases containing information on proteins or ligands are available, and readers who are interested in learning about them can read two further database review papers authored by Zhang Y. et al. (2011) and Moura Barbosa and Del Rio (2012).

### Applications

We reviewed nearly all articles on the applications of shape, pharmacophore screening and reverse docking in searching for the potential targets of small molecules published since 2000 and conducted a systematic analysis. In addition, we have provided slightly more detailed descriptions of some representative examples of the application of existing services to drug discovery. In reviewing these previous studies, we found two approaches to method application: the use of a single method and the use of combined methods. In general, shape screening, pharmacophore screening and reverse docking have all been successfully applied individually. However, these methods have their own limitations in terms of features and application scopes, as mentioned above. The majority of newer examples involve the combined application of multiple methods. Finally, it is noted that all the compounds as examples for illustration in this section are shown in Figure 1.

### Shape screening

Shape-screening services have a wide range of applications. Table 3 shows 18 examples of the use of SEA, TargetHunter, ROCS, SwissTargetPrediction, etc. to perform shape screening for molecular target prediction. Here, even if an article involves several query molecules, we still present them as one example.

**Table 3:** Applications of shape screening in predicting protein targets of small molecules.

**Query molecule**	**Target information**	**Reverse screening tool**	**References**
**EXPERIMENTALLY VERIFIED**			
Prozac^1^, Vadilex^2^, Rescriptor^3^	β1 receptor^1^, 5-HT transporter^2^, HRH4^3^	SEA	Keiser et al., 2009
Wuweizi (compound 11/12)	GBA3^11, 12^, SHBG^11, 12^	SEA	Wang et al., 2015
Lignan	5-HT1AR	SEA	Zheng et al., 2015
Plumbagin	TrxR, GR'	SEA	Hwang et al., 2015
Obacunone	MIF	SEA	Gao et al., 2018
5-aza-dC	HDM2	SuperPred	Putri et al., 2017
Sini decoction (aconitine^1^, liquiritin^2^, 6-gingerol^3^)	ADRB1^1^, ACE^2^, HMGCR^1, 3^	TargetHunter	Zhang H. et al., 2016
Salvinorin A^*^	OPRK, CB1, CB2, DRD2	TargetHunter	Xu et al., 2016b
**NOT EXPERIMENTALLY VERIFIED**			
NBP	NQO1, IDO, NADH-ubiquinone oxidoreductase	SEA	Wang Y. et al., 2017
Quinoline derivative (83b1)	PPARδ	SEA	Pun et al., 2017
Tributyltin (Ch-409)	RamC	SwissTargetPrediction	Waseem et al., 2017
Xeronine	Adr A3, TDP1, muscleblind-like proteins 1	SwissTargetPrediction	Sanni et al., 2017
**KNOWN TARGETS COMPUTATIONALLY VERIFIED**			
Methadone^1^, emetine^2^, loperamide^3^	Muscarinic M3^1^, α2 adrenergic^2^, NK2 receptors^3^	SEA	Keiser et al., 2007
CID 46907796	Nrf2	TargetHunter	Wang L. et al., 2013
Chlorotrianisene	COX-1, ESR1	SwissTargetPrediction	Gfeller et al., 2014
Entecavir	POLB	TarPred	Liu et al., 2015a
Taxol mimetics	Tubulin	CSNAP3D	Lo et al., 2016
Caffeine	D2R	ChemProt	Kringelum et al., 2016

* *Target prediction confirmed by the literature. Superscript values denotes that the protein targets in the second column correspond to the query molecules in the first column respectively*.

Reverse screening based on shape similarity has multiple types of applications. First, it is used to search for the targets of molecules from Chinese herbal medicine. For instance, using TargetHunter, Zhang et al. predicted human beta-1 adrenergic receptor (ADRB1) as the protein target of aconitine (compound **3**), an experimentally active component of Sini Decoction in the treatment of cardiovascular disease, and this prediction has been experimentally verified (Zhang H. et al., 2016). Shape screening is also helpful for drug repositioning and for clarifying the mechanisms of action of existing drugs. For example, Keiser et al. conducted shape screening using SEA to reposition 3,665 FDA-approved and investigational drugs, and they successfully predicted unintended targets of several drugs, such as the antagonism of the β1 receptor by the transporter inhibitor prozac (compound **4**), the inhibition of the 5-HT transporter by the ion channel drug vadilex (compound **5**), and the antagonism of the histamine H4 receptor by the enzyme inhibitor rescriptor (compound **6**; Keiser et al., 2009).

### Pharmacophore screening

Reverse screening based on pharmacophore modeling is also widely used to search for the targets of components of various Chinese traditional medicines. Table 4 shows 27 examples of the use of PharmMapper and Discovery Studio to perform pharmacophore screening for the prediction of molecular targets.

**Table 4:** Applications of pharmacophore screening in predicting protein targets of small molecules.

**Query molecule**	**Target information**	**Reverse screening tools**	**References**
**EXPERIMENTALLY VERIFIED**			
CT	MAP2K1	PharmMapper	Yuan et al., 2014
Arctigenin	PDK1	PharmMapper	Fang et al., 2015
HSYA	XO	PharmMapper	Xu et al., 2016a
ZYZ-488	Apaf-1	PharmMapper	Wang Y. et al., 2016
NCI 748494/1	c-Met kinase	PharmMapper	El-Wakil et al., 2017
UA	CASP-3, JNK2, ERK1	PharmMapper	Ma et al., 2017
BBR	GR, p38, DHODH	PharmMapper	Liu et al., 2017
5,7-dihydroxy-4′-methoxy-8-prenylflavanone	AChE	PharmMapper	Das et al., 2017
Phytoestrogens (genistein^1^, daidzein^2^, secoisolariciresinol^3^)	AKR1B1^1^, H-Ras^2^, GSTP1^3^	PharmMapper	Dutta et al., 2017
**NOT EXPERIMENTALLY VERIFIED**			
MCDF	GR	PharmMapper	Chitrala and Yeguvapalli, 2014
Capsaicin	CA2	PharmMapper	Ye et al., 2015
SID 242078875	DPP-IV, PTP1B, PEPCK, GSK-3B, GK	PharmMapper	Krishnasamy and Muthusamy, 2016
Flavanoid analogs	CDK2	PharmMapper	Simon et al., 2017
Chalcones and chalcone-like compounds	Cysteine proteases	PharmMapper	Gomes et al., 2017
16E-arylideno-nitrogen mustard hybrids(3/4)	GRs	PharmMapper	Acharya et al., 2018
Components of CO	ESR1, ESR2, HSD11B1, cortisone reductase	PharmMapper	Wang N. et al., 2017
N-substituted tetrahydro-β-carboline imidazolium salt derivatives	MEK-1	PharmMapper	Liang et al., 2017
ASC	AKR1B1, ALB, AR, BACE1, CDK2, F2	PharmMapper	Zeng et al., 2017a
GFW compounds	F2, MMP3, CA2, AKR1B, CDK2	PharmMapper	Zeng et al., 2017b
Thiadiazole compounds	c-Met	PharmMapper	Meshram et al., 2017
Components in SFJD	Multi-targets in ERK pathway	PharmMapper	Li et al., 2017
Isoquinoline alkaloids	MIF, ZipA-FtsZ, GAPDH, etc.	Discovery Studio 3.5	Lei et al., 2015
Six GTs	GCN5, CDK2	Discovery Studio 4.0	Shao et al., 2016
Pinctada fucata oligopeptide	5HT2A, BACE-1	Discovery Studio	Chen et al., 2017
**KOWN TARGETS COMPUTATIONALLY VERIFIED**			
Tamoxifen	ERRγ	PharmMapper	Liu et al., 2010
S-adenosyl-L-homocysteine	Modification methylase TaqI	PharmMapper	Wang X. et al., 2016
Kanamycin	APH(2′)-Iva	PharmMapper	Wang X. et al., 2017

^*^ Target prediction confirmed by the literature. Superscript values denotes that the protein targets in the second column correspond to the query molecules in the first column respectively.

For example, Liu et al. used PharmMapper to predict p38, glucocorticoid receptor (GR) and dihydroorotate dehydrogenase (DHODH) as potential targets of berberine (BBR, compound **7**) and further elucidated the possible molecular mechanisms by which these protein targets participate in the anti-melanoma activity of BBR (Liu et al., 2017). Lei et al. employed the Pharmacophore module of Discovery Studio 3.5 in reverse screening and found that the isoquinoline alkaloids (such as compound **8**) extracted from Macleaya cordata (Bo Luo Hui) might target macrophage migration inhibitory factor (MIF), potentially leading to the broad-spectrum antitumor effects of the plant (Lei et al., 2015).

### Reverse docking

Reverse screening based on molecular docking is widely used to search for the targets of small molecules to elucidate their mechanisms of action. Tables 5, 6 show 25 and 20 examples with and without experimental validation, respectively, of the application of reverse docking to the prediction of molecular targets by using TarFisDock, idTarget, INVDOCK, and conventional virtual screening software such as DOCK, MDock, and AutoDock.

**Table 5:** Applications of reverse docking in predicting protein targets of small molecules with experimental verification.

**Query molecule**	**Target information**	**Reverse screening tools**	**References**
Triptonide^1^, triptolide^2^, triptriolide^3^	ERα^1, 2, 3^	AutoDock 4.2	Liu et al., 2015b
c-di-GMP	Human LCN2 protein	DOCK 6	Li et al., 2015
Derivatives of indirubin (6BIO)	PDK1	GlamDock	Zahler et al., 2007
DAPH	Hexokinase	GOLD	Da Matta et al., 2015
Apple polyphenols	GMP reductase, GTPase H-ras, HGPRT	idTarget	Scafuri et al., 2016
Anti-HIV drugs (Pis, NRTIs)^*^	POLB, TOP1, etc.	INVDOCK	Ji et al., 2006
Analgesics^*^	ErbB-2, PLA2, GSH-S	INVDOCK	Pan et al., 2014
GAD	EphA7, EB1, PRDX3	INVDOCK	Yue et al., 2008
BBR	p53	INVDOCK	Lu et al., 2010
SB	EGFR	INVDOCK	Feng et al., 2011
BBR	HSPA8, ANXA5	INVDOCK	Lu et al., 2012
AGS-IV	CN, ACE, JNK	INVDOCK	Zhao et al., 2012
SB^*^	ACE, REN	INVDOCK	Ye et al., 2012
WB	CDK2, PAK4, BRaf1	INVDOCK	Zhang et al., 2013
BBR	CaM	INVDOCK	Ma et al., 2013
Ophiobolin O	GSK3β	INVDOCK	Lv et al., 2015
PRIMA-1	OSC	Mdock	Grinter et al., 2011
Meranzin	COX1, COX2, PPARγ	SELNERGY	Do et al., 2007
Tofisopam	PDE4	SELNERGY	Bernard et al., 2008
Anti-*Helicobacter pylori* drugs (compound 1/2)	HpPDF^1, 2^	TarFisDock	Cai et al., 2006
[6]-gingerol	LTA4H	TarFisDock	Jeong et al., 2009
5 of 19 natural products	DPP-IV	TarFisDock	Zhang S. et al., 2011
Bezafibrate	CDK2	TarFisDock	Liu et al., 2015c
Bicyclol	IMPDHII	TarFisDock	Zhang Y. W. et al., 2016
Esculentoside A	CK2	TarFisDock	Li Y. et al., 2016

* *Target prediction confirmed by the literature. Superscript values denotes that the protein targets in the second column correspond to the query molecules in the first column respectively*.

**Table 6:** Applications of reverse docking in predicting protein targets of small molecules without experimental verification.

**Query molecule**	**Target information**	**Reverse screening tool**	**Reference**
**NOT EXPERIMENTALLY VERIFIED**			
Tea polyphenols	LTA4 hydrolase	Autodock, TarFisDock	Zheng et al., 2011
4 compounds	PBP4	Autodock Vina	Sarangi et al., 2015
Lenalidomide	VEGFR-2, erbB-3, FGFR-4, ABL, p38MAPK, MMP-3	Autodock	Hu et al., 2017
Torcetrapib	PDGFR, HGFR, IL-2, ErbB1	Discovery Studio	Fan et al., 2012
Melamine and cyanuric acid	GPX1, HEXB, LDH, lys C	INVDOCK	Ma et al., 2011
PAs	GSTA1, GPX1	INVDOCK	Yan et al., 2016
Icariin	PI3K, AChE	INVDOCK	Cui et al., 2016
Dioscin	TOP1	MDock	Yin et al., 2015
Ginsenosides	MEK1, EGFR, thrombin, Aurora A	Schrödinger	Park and Cho, 2017
TCDD	MMP8, MMP3, OSC, MPO	TarFisDock	Oliveroverbel et al., 2010
Ganoderic acid	HIV-1 proteasein	TarFisDock	Akbar and Yam, 2011
Fullerene derivatives	HPRT, BACE1	TarFisDock	Gupta et al., 2011
Alpha lipoic acid	LTA4 hydrolase, VGKC	TarFisDock	Maldonado-Rojas, 2011
Aryl-aminopyridine derivatives	CDK2, aurora kinase, KIT receptor	TarFisDock	Erić et al., 2012
**KNOWN TARGETS COMPUTATIONALLY VERIFIED**			
4-HT, vitamin E	ER, GST	INVDOCK	Chen and Zhi, 2001
ASA^1^, gentamicin^2^, ibuprofen^3^, IDV^4^, neomycin^5^, penicillin G^6^, 4-HT^7^, vitamin C^8^	Antithrombin^1^, CA1^2, 5^, SULT1E1^3^, IFABP^4^, GST^6^, ADH^7^, alphaamylase^8^	INVDOCK	Chen and Ung, 2001
Biotin^1^, 4-HT^2^, HDPR^3^, methotrexate^4^	Streptavidin^1^, ERa^2^, ADA^3^, DHFR^4^	GOLD	Paul et al., 2004
ε-viniferin	PDE4	SELNERGY	Do et al., 2005
Vitamin E^1^, 4-HT^2^	AChE^1^, DHFR^2^	TarFisDock	Li et al., 2006
DRV^1^, 6BIO^2^, N-(4-aminobiphenyl-3-yl)-benzamide^3^	HDAC2^1^, HIV-1 PR^2^, CDK2^3^	idTarget	Wang et al., 2012

^*^ Target prediction confirmed by the literature. Superscript values denotes that the protein targets in the second column correspond to the query molecules in the first column respectively.

For example, several research groups used INVDOCK to predict that p53 (Lu et al., 2010), calmodulin (CaM; Ma et al., 2013), annexin A5 (ANXA5) and heat shock protein family A member 8 (HSPA8; Lu et al., 2012) might be protein targets of the broad-spectrum anticancer drug BBR (compound **7**). Zhang et al. employed TarFisDock in the reverse docking of 19 compounds extracted from the traditional Chinese medicines Bacopa monnieri (L.) Wettst and Daphne odora Thunb. Var. Marginata, and they concluded that five of the compounds (such as compound **9**) might target dipeptidyl peptidase IV (DPP-IV), thus accounting for the effectiveness of these medicines in the treatment of diabetes and their anti-inflammatory effects (Zhang S. et al., 2011). Scafuri et al. (2016) applied idTarget to predict that the proteins guanosine triphosphatase (GTPase), guanosine 5′-monophosphate oxidoreductase (GMP reductase) and hypoxanthine-guanine phosphoribosyltransferase (HGPRT) might be key targets of apple polyphenols (such as compound **10** and **11**), resulting in their cancer-preventive effects. Grinter et al. used MDock to fish for the protein targets of the compound PRIMA-1 (compound **12**) in the PDTD database and discovered that PRIMA-1 could inhibit the cholesterol synthetic pathway by directly binding with oxidosqualene cyclase (OSC), considerably reducing the viability of BT-474 and T47-D breast cancer cells (Grinter et al., 2011).

### Hybrid applications

The combinations of methods include shape screening with reverse docking, shape screening with pharmacophore screening, pharmacophore screening with reverse docking, and the combination of all three methods. Table 7 shows 22 examples of the use of these four combinations to predict molecular targets.

**Table 7:** Applications of hybrid screening in predicting protein targets of small molecules.

**Query molecule**	**Target information**	**Method**	**Reverse screening tool**	**References**
**EXPERIMENTALLY VERIFIED**				
Rosemary components (carnosol,CA,UA,RA)^*^	CDK2, MAPK-14, AR', PPARγ	RD&PS	PharmMapper, idTarget	Deshmukh et al., 2013
DIP^*^	DPD, Bub1	RD&PS	PharmMapper, idTarget	Ge et al., 2016
*Magnaporthe oryzae* inhibitor C1	CYP450, NMT, GS, CHS	RD&PS	PharmMapper, TarFisDock	Chen et al., 2016
SAA^*^	AR	RD&PS	DRAR-CPI, PharmMapper	Chen and Cui, 2017
Curcumin	CDK2	RD&SS	Schrödinger	Lim et al., 2014
Naproxen	PI 3-K	RD&SS	Schrödinger	Kim et al., 2014
GV2–20	CA2	RD&SS	ROCS, AutoDock	Mori et al., 2015
Kinetin	Chitinase	RD&SS	idTarget, ReverseScreen3D	Kumar et al., 2015
Glabridin	Braf, MEK1/2	RD&SS	Schrödinger	Wang Z. et al., 2016
Macrocyclic amidinoureas	Chitinase	RD&SS	ROCS, OEDocking	Maccari et al., 2017
α-FMH	GST	PS&SS	PharmMapper, ReverseScreen3D	Considine et al., 2017
Baicalein	COMT, MAO-B	RD&PS&SS	Schrödinger, ReverseScreen3D	Gao et al., 2013
**NOT EXPERIMENTALLY VERIFIED**				
Saffron bioactive ingredients (picrocrocin)	HSP 90-α	RD&PS	PharmMapper, idTarget	Bhattacharjee et al., 2012
Danshensu	GTPase Hras	RD&PS	PharmMapper, idTarget	Chen and Ren, 2014
Tanshinone IIA	RARα	RD&PS	PharmMapper, AutoDock Vina	Chen, 2014
2-thiazolylimino-5-benzylidin-thiazolidin-4-one	COX2, AChE, AR, THRα	RD&PS	PharmMapper, TarFisDock	Iyer et al., 2015
Glycopentalone	CDK-2, VEGFR-2	RD&PS	AutoDock4.2, PharmMapper	Gurung et al., 2016
PGS^1^, PLMF1^2^, 67DiOHC8S^3^	GSTA1^1^, PTPNT1^2, 3^, CBS^3^	RD&PS	PharmMapper, DRAR-CPI	Pereira et al., 2017
Oxindole pentacyclic alkaloids	DHFR, MDM2	RD&SS	TarFisDock, ReverseScreen3D	Kozielewicz et al., 2014
Quercetin	PARP1	RD&SS	SHAFTS, idTarget	Carvalho et al., 2017
Cardamom bioactive components (eucalyptol)	CASP-3, PKA	PS&SS	PharmMapper, ReverseScreen3D	Bhattacharjee and Chatterjee, 2013
Amai alkaloid and pyridine derivatives in maca	AR', CA2, ERα, MAPK14, etc.	PS&SS	Discovery Studio4.5	Yi et al., 2016

* *Target prediction confirmed by the literature. Superscript values denotes that the protein targets in the second column correspond to the query molecules in the first column respectively*.

Eight target prediction examples were performed by combining shape similarity and molecular docking. Kozielewicz et al. employed ReverseScreen3D and TarFisDock to predict the targets of oxindole pentacyclic alkaloids (such as compound **13**) and found that the biological ability of these compounds to induce cancer cell apoptosis may potentially involve the inhibition of several important targets, including dihydrofolate reductase (DHFR) and mouse double minute 2 homolog (MDM2; Kozielewicz et al., 2014). The combination of shape screening and pharmacophore screening has been applied in three instances to predict molecular targets. Biplab Bhattacharjee and Jhinuk Chatterjee used PharmMapper and ReverseScreen3D to perform reverse screening and demonstrated that eucalyptol (compound **14**), the effective component of cardamom, might target CASP-3 and cAMP-dependent protein kinase (PKA), resulting in its anti-apoptosis, anti-inflammation, anti-proliferation, anti-invasion and anti-angiogenesis activities in cancer prevention (Bhattacharjee and Chatterjee, 2013). The combination of pharmacophore modeling and reverse docking has been used most frequently to predict the targets of small molecules, as shown by the 10 applications given in Table 7. For example, Ge et al. used PharmMapper and idTarget in reverse screening and predicted that dihydropyrimidine dehydrogenase (DPD) and human spindle checkpoint kinase Bub1 were the potential unintended or secondary targets of the antithrombotic agent dipyridamole (DIP, compound **15**), resulting in its anti-cancer activity (Ge et al., 2016). Finally, one application combined all three methods for reverse screening. Gao et al. used the Pharmacophore Modeling and Docking modules in Schrödinger as well as RerverseScreen3D jointly to predict molecular targets and found that baicalein (compound **16**), an anti-Parkinson disease drug, played a protective role in the nervous system by targeting catechol-O-methyltransferase (COMT) and monoamine oxidase B (MAO-B); these targets were confirmed experimentally (Gao et al., 2013).

## Discussion

### Comparison of the applications of the three types of reverse screening software and online services in the prediction of small-molecule targets

**Figure 5:**
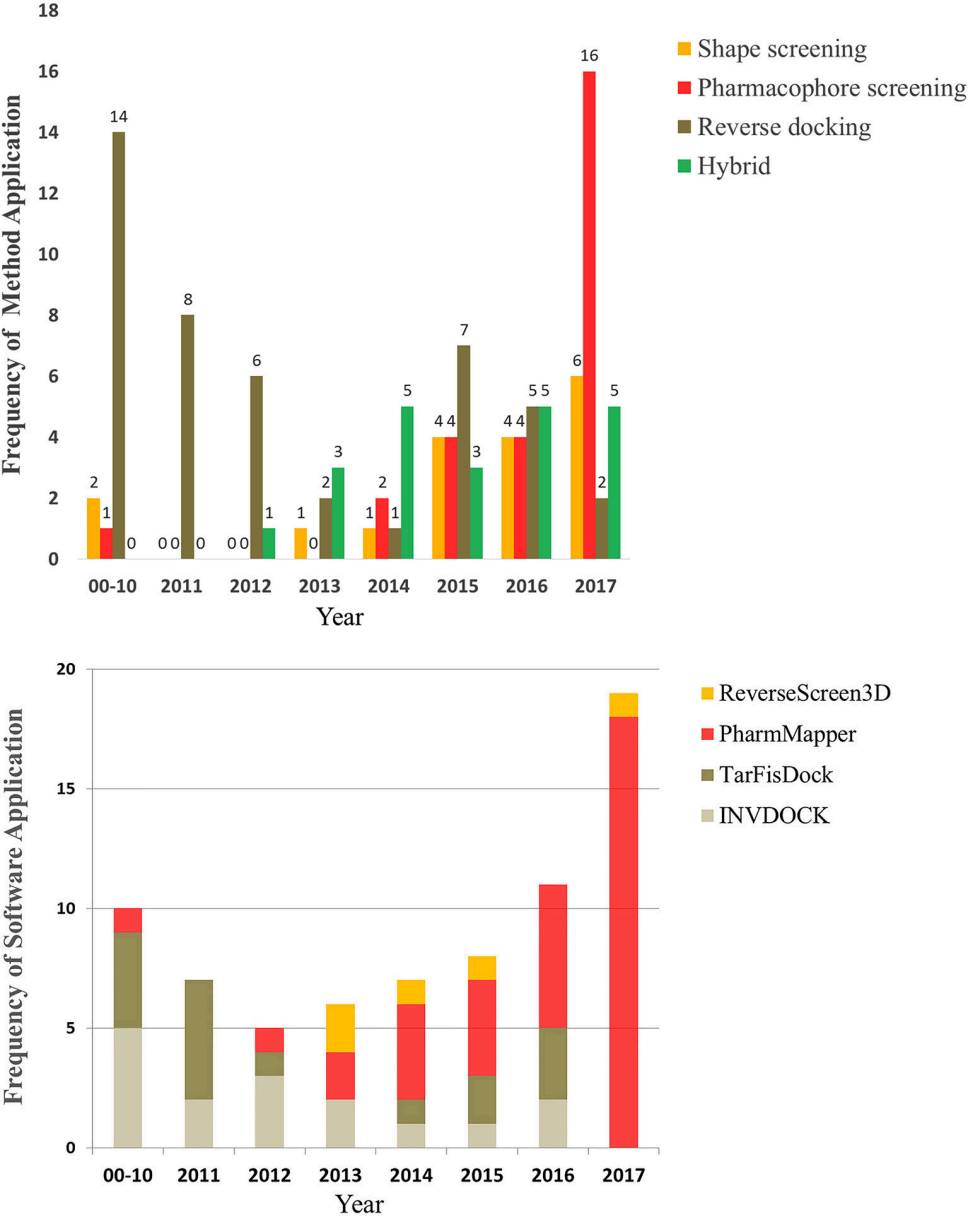
The number and trend of applications using the three reverse screening methods and representative software since the year 2000.

To provide a better understanding of the application of reverse screening for small-molecule target prediction, we collected as many reverse screening tools and databases as possible, most of which have been updated since 2016 (Tables 1, 2). In addition, we counted the number of applications of these three methods since 2000, the number of applications of their representative software programs, and the application trends over the years. All information is shown in Tables 3–7 and Figures 5A,B.

Different types of reverse screening tools have characteristic features. Many software programs and online services are based on shape similarity, and they have a rich supply of ligand databases. These shape screening tools have rapidly updated in-house databases, can screen large chemical databases rapidly, and therefore can be used for large-scale preliminary reverse screening. Although shape-screening methods have the smallest number of applications, a slow upward trend is evident in recent years (Figure 5A). Notably, few studies have applied TarPred, SwissSimilarity, and ChemMapper. The main software and online services based on pharmacophore modeling are PharmMapper and Discovery Studio, each with its own pharmacophore database. PharmMapper is a free online service, whereas Discovery Studio is commercial software, leading to more widespread use of PharmMapper. Among the three reverse screening methods, pharmacophore modeling has the fewest applications before 2016 (Figures 5A,B), but its application exhibited a significantly escalating trend in 2017 (Figures 5A,B). The reverse docking method has been applied the most; however, the applications of reverse docking have shown a downward trend in recent years. The possible reasons are that some online services, such as TarFisDock, have undergone limited expansion of the existing protein crystal structure grid database, while others, such as idTarget, have a long computational time and high computational cost. Figure 5A also illustrates the trend of the practical applications of hybrid methods, with a slow rise in the use of combinations of multiple reverse screening methods for target prediction in recent years.

**Figure 6:**
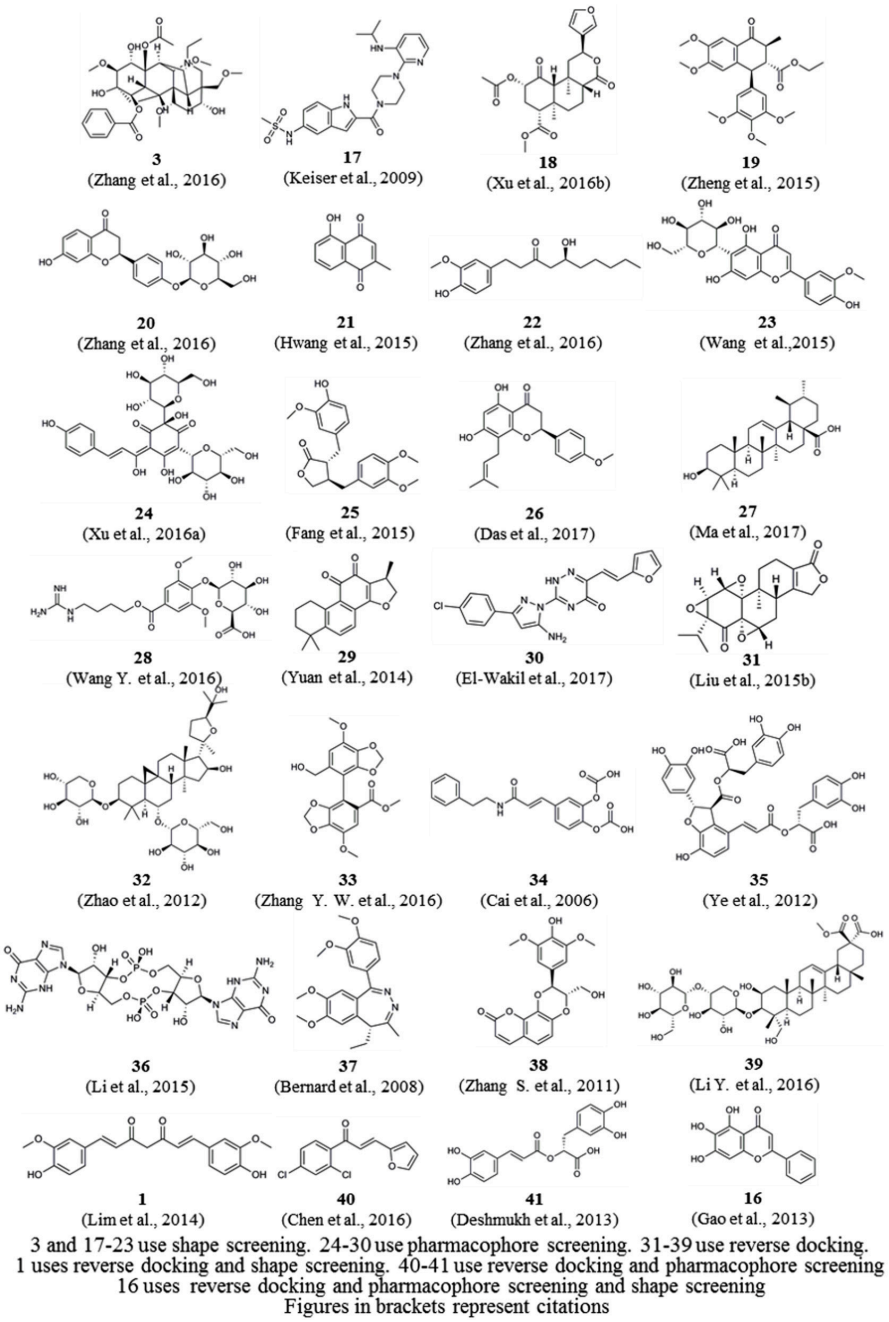
Twenty-eight representative compounds obtained by the clustering of 57 bioactive compounds for target prediction by different reverse screening methods.

In addition, we downloaded from PubChem or sketched in Schrödinger the structures of 57 small-molecule ligands whose targets were predicted by reverse screening and further verified experimentally, as reported in these application articles. We used the Cluster Analysis Module in Schrödinger via the two-tiered drop-down menu of Maestro's Scripts/Cheminformatics/Clustering of Ligands and a pop-up window titled Clustering based on Volume Overlap to conduct a cluster analysis of these small molecules. Figure 6 shows representative compounds in the 28 clusters we obtained. Their targets were predicted separately by shape screening, pharmacophore screening and reverse docking. By comparing the structures of these molecules, we may be able to summarize some rules regarding the application ranges of each type of method according to the structures of the query molecules. For example, shape screening may be suitable for a query molecule whose structure shows stereoscopic sense even in a two-dimensional structural view and that is neither very large nor very small (Compounds **3** and **17–23**). Pharmacophore screening is appropriate for query molecules whose structures contain diverse pharmacophore functional groups with a good balance between them (compounds **24–30**). Reverse docking is suitable for the most diverse range of query molecules (compounds **31–39**). We hope that this type of cluster analysis can provide some guidance for the effective use of reverse screening to predict small-molecule targets in the future.

### Deficiencies in current reverse screening methods and potential solutions

Each reverse screening tool has its own characteristics and appropriate application scope in terms of principle, algorithm and program. However, we need to know the application scopes of these tools in order to select the most appropriate software for making accurate predictions. Thus, a horizontal comparison can provide a better understanding of the advantages and disadvantages of these reverse screening tools and their in-house databases. Some clear deficiencies are present in the programs and in-house databases of current reverse screening tools, making a comparison of the efficiency or the accuracy of target prediction by these tools difficult. None of the online services has a general interface module that can be used to upload and recognize user databases. Because these tools cannot use external databases, evaluating the methods or services based on benchmark databases is infeasible. However, researchers may be able to test the pros and cons of these tools in a way that does not require a benchmark database: we may not need to know the superiority of these tools over each other but may instead need to learn their practical uses and application scopes so that they can be better applied in real-life practice. This comparison requires studies to select some benchmarking query compounds whose known targets represent a large category and whose secondary targets or non-targets have also been studied thoroughly. We can use evaluation indexes such as the enrichment factor and receiver operating characteristic (ROC) curve (Truchon and Bayly, 2007) to assess the practical effects of reverse screening tools on the prediction of targets within this large category for other small molecules, thus achieving a horizontal comparison of existing software and online services. This theory is not yet perfected, and successful examples of this approach remain lacking, but it may provide prospects for developing assessments of reverse screening methods and tools.

Moreover, reverse screening servers also lack general-type databases, and their in-house databases are not publicized. We cannot learn the inclusion and exclusion criteria for building these direct databases. Almost all direct databases are bound to the corresponding software, and we are unable to conduct potential data mining. The resources of different online services are also undisclosed, and the services cannot refer to each other's databases. Hence, we encourage the developers of all software and online services to disclose their own databases and their construction processes to facilitate user comprehension and utilization. Only in this way can these software databases be better applied in practice, and this approach could also promote the production of more excellent protein-annotated ligand or target structure grid databases for reverse screening.

### Previous reviews and prospective studies on reverse screening in molecular target prediction

To date, five reviews of reverse screening are available in the literature, which we will discuss briefly below. Readers can also peruse these reviews to deepen their understanding of molecule target prediction algorithms. We will not address other reviews that involve the use of experimental methods or a combination of computational and experimental methods to predict molecular targets (Schenone et al., 2013).

Three of the five reviews are similar to our work and provide broad overviews of in silico target fishing. They describe the principles, databases and software involved in computer-aided small-molecule target prediction in terms of different aspects, perspectives and levels (Rognan, 2010; Zheng et al., 2013; Cereto-Massagué et al., 2015). Cereto-Massagué et al. (2015) categorize the methods of target fishing into four classes according to computational principles: molecular similarity methods, protein structure-based methods, data mining/machine learning methods, and methods based on the analysis of bioactivity spectra. Our review covers the principles and applications of the first two classes, molecular similarity and protein structure, but does not address the latter two categories of machine learning and bioactivity spectra. Therefore, readers can review the article by Cereto-Massagué et al. carefully if they are interested in the calculation methods used in those latter two categories. Rognan et al. (Rognan, 2010) describe only protein structure-based approaches and further classify them into protein-ligand docking, structure-based pharmacophore searches, binding site similarity measurements, and protein-ligand fingerprints. Based on the principle of receptor structure-based screening, the authors describe these four sub-methods in detail and discuss their pros and cons for target fishing and ligand profiling. That review features descriptions of protein pocket similarity matching and the molecular fingerprinting of protein-ligand interaction information, which is worth reading and comparing to our paper. In addition, Zheng et al. (2013) provide a comprehensive overview of computer-aided drug design methods, including conventional (forward) virtual screening and reverse screening, in terms of five aspects: drug target prediction, drug repositioning, protein-ligand interaction, virtual screening and lead optimization, and ADME/T (absorption, distribution, metabolism, excretion, and toxicity) property prediction. The three reverse screening methods we reviewed are closely related to drug target prediction, drug repositioning and protein-ligand interaction. The above review can help readers systematically study and understand the field of computer-aided virtual screening in drug design.

The other two reviews address only reverse docking and its applications. Specifically, Kharkar et al. (2014) give a detailed description of reverse docking programs and their target databases and further discuss the applications of reverse docking in target identification and the prediction of target functions and off-target effects. Lee et al. (2016) summarize target databases, software programs and services and discuss the application of reverse docking in small-molecule target recognition and drug discovery. They also professionally discuss four issues related to reverse docking that remain to be solved: the standardization of database construction, the inclusion of receptor flexibility, the time-consuming nature of flexible receptor docking, and the inaccuracy of binding free energy calculations and ligand binding pose prediction. Readers may refer to these two reviews for a more comprehensive understanding of reverse docking methods.

## Conclusion

In this review article, based on previous studies, we selected the three most commonly used types of reverse screening methods, i.e., methods based on shape similarity, pharmacophore modeling and molecular docking, and provided a detailed and comprehensive introduction, including a description of the principles underlying each method and a systematic classification of software, online services, and databases. In addition, we collected nearly all the articles related to the application of computer-aided target reverse screening prediction published since 2000 and analyzed the possible relationships or correlations between compound structures and screening methods by using cluster analysis. The purpose of this review is to help readers quickly understand these three methods and the characteristics of the software and online services based on these methods, to familiarize readers with the status and applications of the different levels of ligand and protein databases used in reverse screening and to provide a better understanding of how existing tools can be applied to molecule target prediction. We strongly believe that more accurate predictions resulting from the familiarity of users with the existing online services and databases will increase the importance of reverse screening in drug repositioning and future research on the pharmacodynamics and pharmacological mechanisms of bioactive compounds.

## Author contributions

HH, GZ, YZ, CL, SC, YL, and SM: Information retrieval and analysis and reverse tools identification and classification; HH, GZ, LC, YL, and ZH: Database classification; HH, GZ, YZ, and ZH: Reference classification; HH, GZ, YZ, LC, SC, YL, SM, and ZH: Manuscript writing; HH, GZ, and ZH: Manuscript revision; HH, GZ, SC, YL, and ZH: Figure processing and table processing; HH, GZ, CL, and ZH: Cluster analysis; ZH: Manuscript guidance, communication work, and financial support.

### Conflict of interest statement

The authors declare that the research was conducted in the absence of any commercial or financial relationships that could be construed as a potential conflict of interest.

## Abbreviations

4-HT: 4-Hydroxy-tamoxifen
5-Aza-dC: 5′-Aza-2′-deoxycytidine
5-HT1AR: 5-Hydroxytryptamine 1A receptor
5-HT2A: 5-Hydroxytryptamine receptor 2A
67DiOHC8S: 6,7-Dihydroxycoumarin-8-sulfate
6BIO: 6-Bromo-indirubin-3′oxime
ABL: BCR-ABL tyrosine kinase
ACE: Angiotensin converting enzyme
ACHE: Acetylcholinesterase
ACM2: Muscarinic acetylcholine receptor 2
ADA: Adenosine deaminase
ADH: Alcohol dehydrogenase
AdrA3: Adenosine receptors A3
ADRB1: Human beta-1 adrenergic receptor
AGS-IV: Astragaloside IV
AKR1B1: Aldo-keto reductase family 1, member B1
ANXA5: Annexin A5
Apaf-1: Apoptotic protease activating factor-1
APH(2′)-Iva: Aminoglycoside-2′-phosphotransferase type Iva
AR: Aldose reductase
AR′: Androgen receptor
ASA: Aspirin
BACE1, ASC: Astragalus Salvia compound, Beta-secretase 1
BBR: Berberine
Braf: B-raf kinase
Bub1: Human spindle checkpoint kinase Bub1
CA: Carnosic acid
CA1: Carbonic anhydrase 1
CA2: Carbonic anhydrase 2
CaM: Calmodulin
CASP-3: Cysteinyl aspartate specific proteinase 3
CB1: Cannabinoid receptor 1
CB2: Cannabinoid receptor 2
CBS: Cystathionine beta-synthase
c-di-GMP: Cyclic diguanylate monophosphate
CDK2: Cyclin dependent kinase-2
CHS: Chitin synthase
CK2: Casein kinase 2
CN: Calcineurin
CO: Curculigo orchioides
Complex I: NADH:ubiquinone oxidoreductase
COMT: Catechol-O-methyltransferase
COX-1: Prostaglandin G/H synthase 1
COX1: Cyclooxygenase-1
COX2: Cyclooxygenase-2
CT: Cryptotanshinone
CYP450: Cytochrome p450
D2R: Dopamine D2 receptor
DAPDC: Diaminopimelate decarboxylase
DAPH: Dialkylphosphorylhydrazone
DHFR: Dihydrofolate reductase
DHODH: Dihydroorotate dehydrogenase
DIP: Dipyridamole
DPD: Dihydropyrimidine dehydrogenase
DPP-IV: Dipeptidyl peptidase IV
DRD2: Dopamine receptor D2
EB1: Microtubule-associated protein RP/EB family member 1
EGFR: Epidermal growth factor receptor
EphA7: Ephrin receptor EphA7
ErbB-1: ErbB-1 tyrosine kinase
ErbB-2: ErbB-2 tyrosine kinase
ERK1: Extracellular regulated protein kinases 1
ERR-γ: Estrogen-related receptor-γ
ERα: Human estrogen receptor alpha
ESR1: Estrogen receptor alpha
ESR2: Estrogen receptor beta
FAK: Focal adhesion kinase
FGFR-4: Fibroblast growth factor receptor 4
GAD: Ganoderic acid D
GAPDH: Glyceraldehyde-3-phosphate Dehydrogenase
GBA3: Cytosolic beta-glucosidase
GCN5: General control non-derepressible 5
GCR: Glucocorticoid receptor
GFW: Guizhi Fuling Wan
GK: Glucokinase
GMP reductase: Guanosine 5′-monophosphate oxidoreductase
GPX1: Glutathione peroxidase 1
GR: Glucocorticoid receptor
GR′: Glutathione reductase
GS, β(1: 3)-Glucan synthase
GSH-S: Glutathione synthetase
GSK3β: Glycogen synthase kinase-3 beta
GST: Glutathione S-transferase
GSTA1: Glutathione S-transferase A1
GSTP1: Glutathione s-transferase PI-1
GTPase: Guanosine triphosphatase
GTs: Ganoderma triterpenes
HDAC2: Histone deacetylase 2
HDPR: 6-hydroxyl-1,6-dihydropurine ribonucleoside
HEXB: beta-Hexosaminidase
HGFR: Hepatocyte Growth Factor Receptor
HGPRT: Hypoxanthine-guanine phosphoribosyltransferase
HIV-1 PR: HIV-1 protease
HMGCR: 3-Hydroxy-3-methylglutaryl-coenzyme A reductase
HpPDF: H. pylori PDF
HPRT: Hypoxanthine phosphoribosyltransferase
HRAS: Harvey rat sarcoma
HRH4: Histamine receptor H4
HSD11B1: 11 beta-Hydroxysteroid dehydrogenase type 1
HSPA8: Heat shock protein family A member 8
HSYA: Hydroxysafflor yellow A
IDO: Indoleamine 2,3-dioxygenase
IDV: Indinavir
I-FABP: Intestinal fatty acid binding protein
IGF1-R: Insulin-like growth factor 1 receptor
IL-2: Interleukin-2
IMPDHII: Inosine 5′-monophosphate dehydrogenase II
JNK: c-Jun N-terminal kinase
LCN-2: Lipocalin-2
LDH: L-lactate dehydrogenase
LTA4H: Leukotriene A4 hydrolase
lysC: Lysozyme C
MAO-B: Monoamine oxidase B
MAP2K1: Mitogen-activated protein kinase kinase 1
MAPK-14: Mitogen-activated protein kinase 14
MCDF: 6-Methyl-1,3,8-trichlorodibenzofuran
MDM2: Mouse double minute 2 homolog
MEK1: Mitogen-activated protein kinase 1
MIF: Migration inhibitory factor
MMP3: Metalloproteinase 3
MMP8: Metalloproteinase 8
MPO: Myeloperoxidase
MTX: Methotrexate
NBP: DL-3-n-Butylphthalide
NF-kB: Nuclear factor kB
NK2 receptor: Neurokinin NK2 receptors
NMT: N-myristoyltransferase
NQO1: NAD(P)H quinone oxidoreductases
Nrf2: Nuclear factor erythroid 2-related factor 2
OBA: Obacunone
OPRK: Kappa opioid receptor
OSC: Oxidosqualene cyclase
p38 MAPK: p38 Mitogen-activated protein kinase
PARP1: Poly [ADP-ribose] polymerase 1
PAs: Pyrrolizidine alkaloids
PBP4: Penicillin binding protein 4
PDE4: Phosphodiesterase 4
PDF: Peptide deformylase
PDGFR: Platelet-Derived Growth Factor Receptor
PDK1: Phosphoinositide-dependent kinase-1
PEPCK: Phosphoenolpyruvate carboxykinase
PGS: Phenolic acid glycoside sulfate
PI-3K: Phosphoinositide 3-kinase
PKA: cAMP-dependent protein kinase
PLA2: Phospholipase A2
PLMF1: Periodic leaf movement factor 1
POLB: DNA polymerase beta
PPARγ: Peroxisome proliferator-activated receptor γ
PPARδ: Peroxisome proliferator-activated receptor delta
PRDX3: Thioredoxin-dependent peroxide reductase mitochondrial precursor
PRIMA-1: P53 reactivation and induction of massive apoptosis
PTP1B: Protein tyrosine phosphatase 1B
PTPNT1: Protein tyrosine phosphatase non-receptor type 1
RA: Rosmarinic acid
RARα: Retinoic acid receptor alpha
REN: Renin
SAA: Salvianolic acid A
SB: Salvianolic acid B
SFJD: Shufengjiedu Capsule
SHBG: Sex hormone-binding globulin
SND: Sini decoction
STAT3: Signal transducer and activator of transcription 3
SULT1E1: Estrogen sulfotransferase
TCDD: 2,3,7,8-tetrachlorodibenzo-p-dioxin
TDP1: Tyrosyl-DNA phosphodiesterase 1
THRa: Human thyroid hormone receptor alpha
TOP1: DNA topoisomerase 1
UA: Ursolic acid
TrxR: Thioredoxin reductase
VEGFR-2: Vascular endothelial growth factor receptor
VGKC: Voltage gated potassium channel
WB: Wentilactone B
XO: Xanthine oxidase.
